# Supplementary material for: Insular cortical circuits as an executive gateway to decipher threat or extinction memory via distinct subcortical pathways
Source: Nat Commun. 2022 Sep 21;13:5540. doi: 10.1038/s41467-022-33241-9 (PMC9492683; doi:10.1038/s41467-022-33241-9)
Supplement: Supplementary file 1 — Supplementary Information [file 41467_2022_33241_MOESM1_ESM.pdf]

Supplementary Information for

Insular Cortical Circuits as an Executive Gateway to Decipher Threat or Extinction Memory via Distinct Subcortical Pathways

Qi Wang[1,2,8], Jia-Jie Zhu[2,8], Lizhao Wang[2], Yan-Peng Kan[2], Yan-Mei Liu[1,2], Yan-Jiao Wu[1,2], Xue Gu[1,2], Xin Yi[1–3], Ze-Jie Lin[1,2], Qin Wang[2], Jian-Fei Lu[1,2], Qin Jiang[1,2], Ying Li[1,2], Ming-Gang Liu[1,2], Nan-Jie Xu[2], Michael X. Zhu[4], Lu-Yang Wang[5,6], Siyu Zhang[1,2,7,*], Wei-Guang Li[1–3,7*], Tian-Le Xu[1,2,7,*]

[1]Center for Brain Science, Shanghai Children's Medical Center, Shanghai Jiao Tong University School of Medicine, Shanghai 200127, China;
[2]Department of Anatomy and Physiology, Shanghai Jiao Tong University School of Medicine, Shanghai 200025, China;
[3]Department of Rehabilitation Medicine, Huashan Hospital, Institute for Translational Brain Research, State Key Laboratory of Medical Neurobiology and Ministry of Education Frontiers Center for Brain Science, Fudan University, Shanghai 200032, China;
[4]Department of Integrative Biology and Pharmacology, McGovern Medical School, University of Texas Health Science Center at Houston, Houston, TX 77030, USA;
[5]Program in Neuroscience and Mental Health, SickKids Research Institute, Toronto M5G 1X8, Canada;
[6]Department of Physiology, University of Toronto, Toronto M5S 1A1, Canada;
[7]Shanghai Research Center for Brain Science and Brain-Inspired Intelligence, Shanghai 201210, China;
[8]These authors contributed equally.

[*]Corresponding author:
Tian-Le Xu, PhD (E-mail: xu-happiness@shsmu.edu.cn)
Wei-Guang Li, PhD (E-mail: liwg@fudan.edu.cn)
Siyu Zhang, PhD (E-mail: zhang_siyu@sjtu.edu.cn)

Supplementary Information provided in this file:

Supplementary Fig. 1. Laminar distribution of FosTRAPed memory ensembles in anterior IC.
Supplementary Fig. 2. Correlation of FosTRAPed IC fear- and extinction-memory ensembles and freezing level.

Supplementary Fig. 3. Functional validation of IC fear- and extinction-memory ensembles.
Supplementary Fig. 4. Characterization of segregation of fear- and extinction-memory ensembles in IC.
Supplementary Fig. 5. Largely non-overlapping IC neuronal subpopulations differentially project to the CeA and NAc.
Supplementary Fig. 6. Histological verification of GCaMP6m expression and optical fiber placements for recording calcium response of IC-CeA (left) and IC-NAc (right) projectors, related to **Fig. 4**.
Supplementary Fig. 7. Z-score analysis of response patterns of IC-CeA and IC-NAc projectors to fear learning and fear- or extinction-memory retrieval.
Supplementary Fig. 8. Quantification of response patterns of IC-CeA and IC-NAc projectors to fear learning and fear- or extinction-memory retrieval by analyzing the initial responses to tone or shock.
Supplementary Fig. 9. Lack of fluorescence change in IC-CeA and IC-NAc projectors expressing mCherry to fear learning and fear- or extinction-memory retrieval.
Supplementary Fig. 10. No effect of genetic silencing of IC-CeA or IC-NAc projectors on locomotor activity, related to **Fig. 5a–d**.
Supplementary Fig. 11. Effects of optogenetic inhibition of IC-CeA or IC-NAc projectors on fear and extinction memories, respectively.
Supplementary Fig. 12. Effects of optogenetic inhibition of IC-CeA or IC-NAc projectors on fear and extinction memory retrieval, respectively.
Supplementary Fig. 13. No effect of optogenetic inhibition of IC-CeA or IC-NAc projectors on locomotor activity.
Supplementary Fig. 14. Histological and electrophysiological verifications of optogenetic activation of IC-CeA or IC-NAc projectors.
Supplementary Fig. 15. Effects of optogenetic activation of IC-CeA projectors on unconditioned fear behaviors or conditioned fear behaviors induced by strong conditioning protocols.
Supplementary Fig. 16. Effects of optogenetic activation of IC-NAc projectors on fear conditioning and subsequent memory retrieval.
Supplementary Fig. 17. No effect of optogenetic activation of IC-CeA or IC-NAc projectors on locomotor activity.
Supplementary Fig. 18. IC-CeA or IC-NAc projectors contribute to fear- or extinction-memory-associated conditioned place aversion or preference, respectively.
Supplementary Fig. 19. Quantification of electrophysiological properties of separate IC-CeA and IC-NAc projectors.
Supplementary Fig. 20. Electrophysiological characterization of synaptic projections from the IC-CeA projector to the IC-NAc projector and vice versa.
Supplementary Fig. 21. Characterization of starter cells for RV-mediated retrograde tracing of IC-CeA or IC-NAc projectors.
Supplementary Fig. 22. Quantification of labeled monosynaptic inputs to IC-CeA or IC-NAc projectors.
Supplementary Fig. 23. Electrophysiological verification of biased projections from

the OFC to IC-NAc or IC-CeA projectors.

Supplementary Fig. 24. Histological confirmation of optical fiber placements for optogenetic activation of OFC→IC projection or OFC→IC→NAc pathway, related to **Fig. 7c–g**.

Supplementary Fig. 25. Histological and electrophysiological verifications of ChR2-mCherry expression in IC-NAc projectors dedicated to receiving long-range projections from the OFC.

Supplementary Fig. 26. Scheme for cortical circuits as an executive gateway to decipher threat or extinction memory via distinct subcortical pathways.

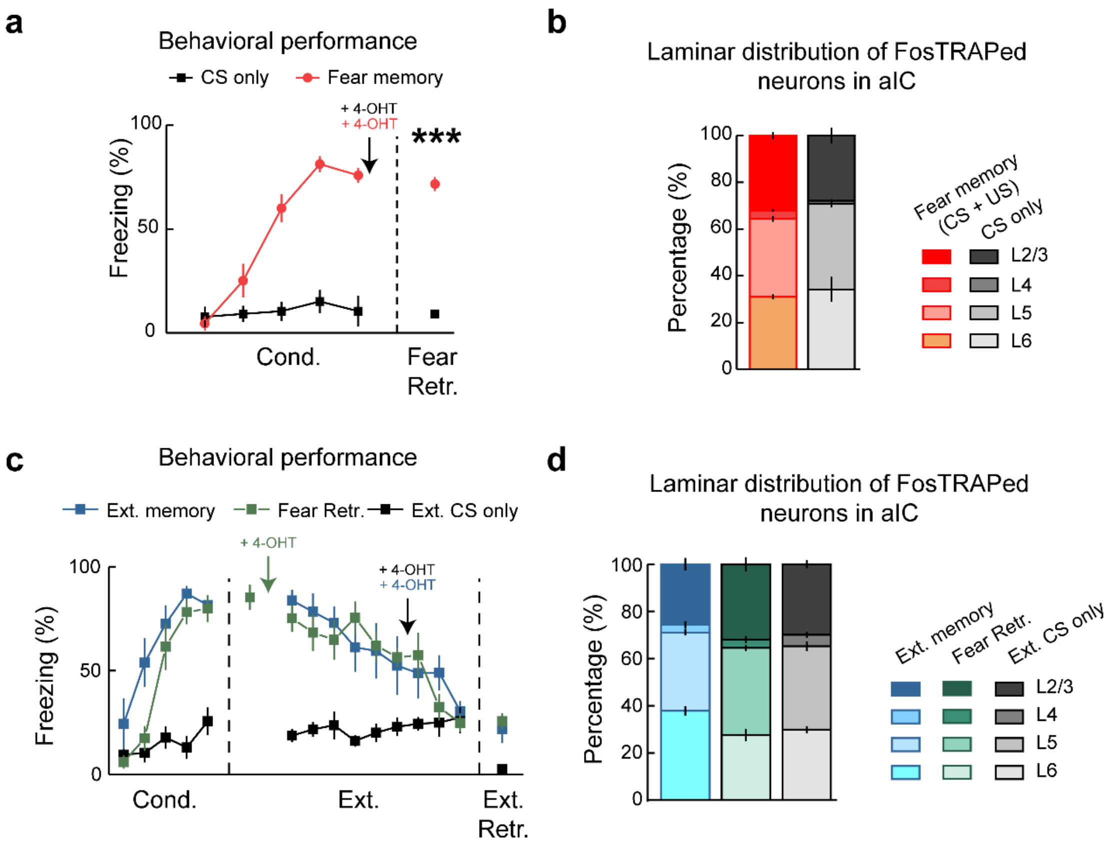

Supplementary Fig. 1. Laminar distribution of FosTRAPed memory ensembles in anterior IC. a Behavioral performance of FosTRAP2 mice labeling fear memory ensembles. Freezing responses to the CS during fear conditioning (Cond.), fear-memory retrieval (Fear Retr.), CS only, n = 8 mice; Fear memory, n = 8 mice. Cond.: $F_{(1, 14)} = 50.31$, $^{***}P = 5.3964\text{E}{-}04$, two-way repeated measures ANOVA; Fear Retr., $t_{(14)} = 17.5$, $^{***}P = 6.5174\text{E}{-}11$, two-tailed unpaired Student's *t*-test. **b** Laminar distribution of FosTRAPed memory ensembles in anterior IC. Fear memory, n = 5 mice; CS only, n = 4 mice. **c** Behavioral performance of FosTRAP2 mice labeling extinction memory ensembles. Freezing responses to the CS during fear conditioning (Cond.), extinction training (Ext.), and subsequent memory retrieval (Ext. Retr.). Ext. memory, n = 6 mice; Fear Retr., n = 7 mice; Ext. CS only, n = 7 mice. Cond.: $F_{(2, 17)} = 29.37$, $^{***}P = 3.0518\text{E}{-}06$, two-way repeated measures ANOVA, main effect of groups, followed by Bonferroni's test, $^{*}P = 0.0144$ for Ext. memory vs. Fear Retr., $^{***}P < 0.001$ for Ext. memory vs. Ext. CS only; $^{***}P < 0.001$ for Fear Retr. vs. Ext. CS only; Ext.: $F_{(2, 17)} = 16.1$, $^{***}P = 1.1943\text{E}{-}04$, two-way repeated measures ANOVA, main effect of AAV, followed by Bonferroni's test, $P > 0.9999$ for Ext. memory vs. Fear Retr., $^{***}P < 0.001$ for Ext. memory vs. Ext. CS only, $^{***}P < 0.001$ for Fear Retr. vs. Ext. CS only; Ext. Retr.: $F_{(2, 17)} = 8.603$, $^{**}P = 0.0026$, one-way ANOVA, main effect of AAV, followed by Student–Newman–Keuls test, N.S. for Ext. memory vs. No. Ext, $^{**}P < 0.01$ for Ext. memory vs. Ext. CS only. $^{**}P < 0.01$ for Fear Retr. vs. Ext. CS only. **d** Laminar distribution of FosTRAPed memory ensembles in anterior IC. Ext. memory, n = 5 mice;

No. Ext. n = 7 mice; CS only, n = 4 mice. Data are presented as mean values ± SEM and the error bar represents SEM. Source data are provided as a Source Data file.

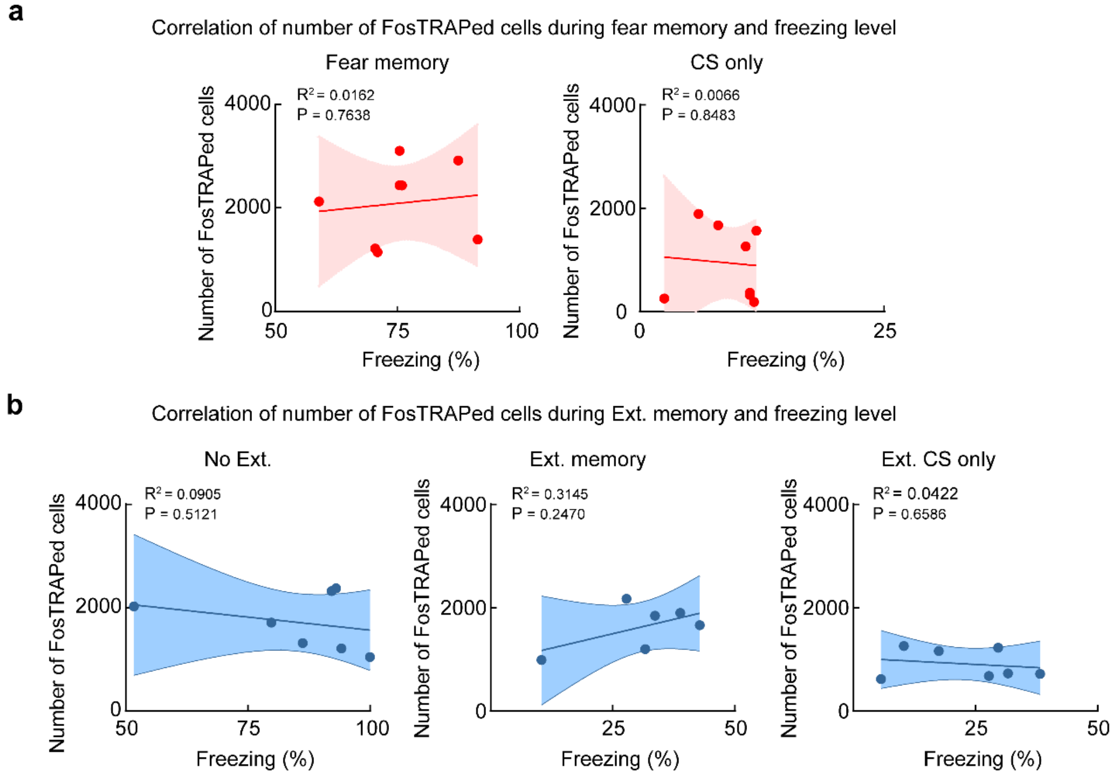

Supplementary Fig. 2. Correlation of FosTRAPed IC fear- and extinction-memory ensembles and freezing level. a Correlation of number of FosTRAPed cells during fear memory and the level of behavioral freezing. Data are from Fig. 1. **b** Correlation of number of FosTRAPed cells during extinction memory and the level of behavioral freezing. Data are from Fig. 2. Pearson coefficient (R) and P values were indicated for each plot.

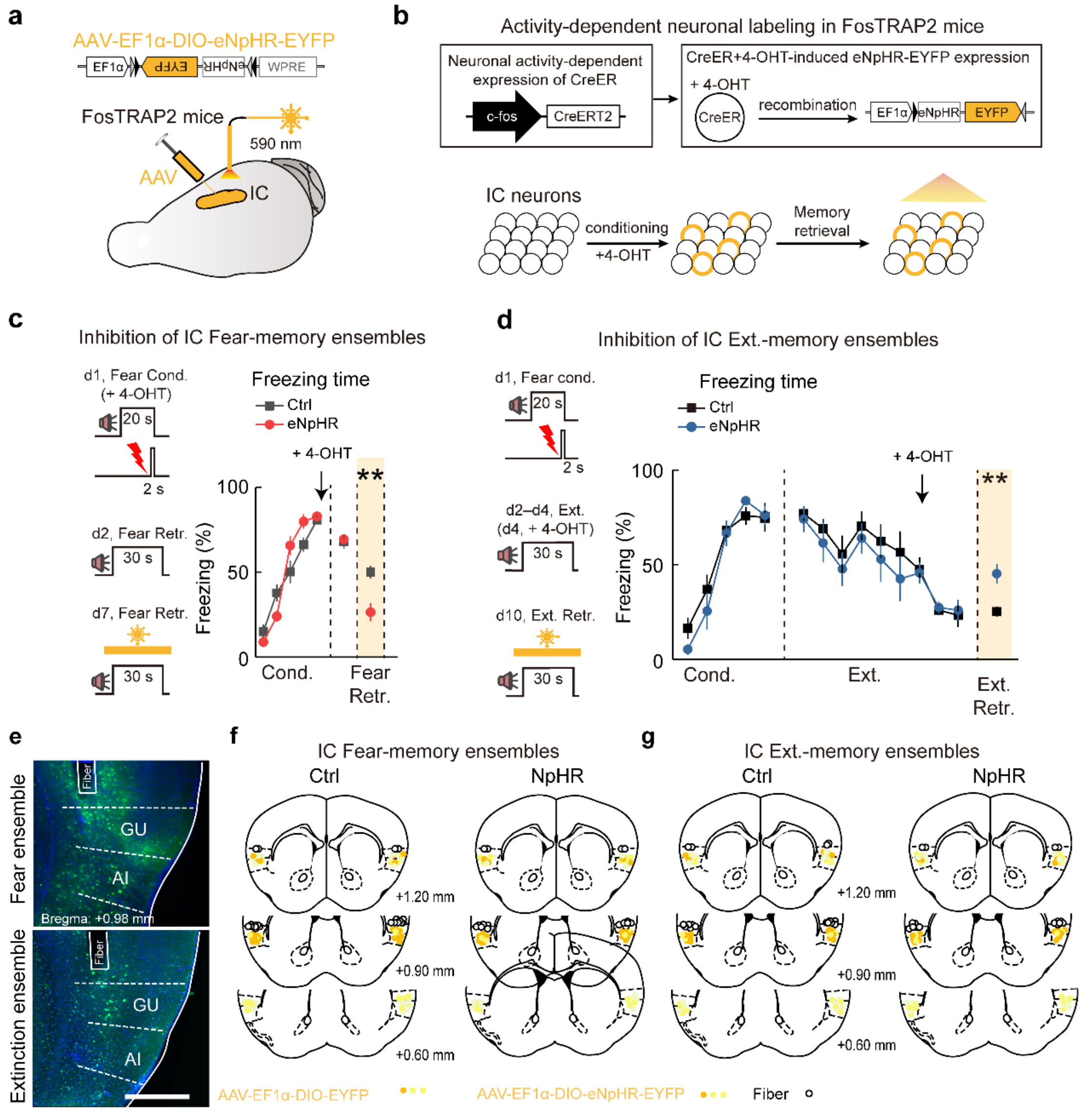

Supplementary Fig. 3. Functional validation of IC fear- and extinction-memory ensembles. **a** Schematic of AAV injections. **b** Experimental design. Yellow circles, FosTRAPed neurons activated during behavioral tagging. **c** Effects of optogenetic inhibition of IC fear memory ensembles. Left: behavioral protocols. Right: behavioral responses. The yellow-shadowed box denotes the light-on phase. Freezing responses to the CS during fear conditioning and memory retrieval with or without yellow light. While the freezing responses during conditioning were calculated by the freezing to every CS per trial, those during memory retrieval were calculated by the average freezing responses during every four trials. Ctrl (EYFP), n = 10 mice; eNpHR, n = 8 mice. Cond.: $F_{(1, 16)} = 0.3673$, $P = 0.5530$, two-way repeated measures ANOVA; Fear Retr., without light: $t_{(16)} = 0.2685$, $P = 0.7917$; Fear Retr., with light: $t_{(16)} = 3.716$, $^{**}P = 0.0019$, two-tailed unpaired Student's *t*-test. **d** Effects of optogenetic inhibition of IC extinction memory ensembles. Left: behavioral protocols. Right: behavioral responses. The yellow-shadowed box denotes the light-on phase. Freezing responses to the CS

during fear conditioning (Cond.), extinction training (Ext.), and memory retrieval (Ext. Retr.). Ctrl (EYFP), n = 8 mice; eNpHR, n = 8 mice. Cond.: $F_{(1, 14)} = 0.3051$, $P = 0.5894$; Ext.: $F_{(1, 14)} = 0.4695$, $P = 0.5044$, two-way repeated-measures ANOVA. Ext. Retr. with light: $t_{(14)} = 3.613$, $^{**}P = 0.0028$, two-tailed unpaired Student's *t*-test. **e** Histological verification of eNpHR-EYFP expression in IC fear or extinction memory ensembles. Representative images of Cre-ERT2 expression (EYFP) in a FosTRAP2 mouse that received AAV-EF1α-DIO-eNpHR-EYFP (or -EYFP as Ctrl) injection into the IC. Scale bar, 500 μm. **f, g** Histological confirmation of optical fiber placements for optogenetic inhibition of IC fear (**f**) or extinction (**g**) memory ensembles. **f** Fear memory ensembles, Ctrl, n = 10 mice; eNpHR, n = 8 mice. **g** Ext. memory ensembles, Ctrl, n = 8 mice; eNpHR, n = 8 mice. Data are presented as mean values ± SEM and the error bar represents SEM. Source data are provided as a Source Data file.

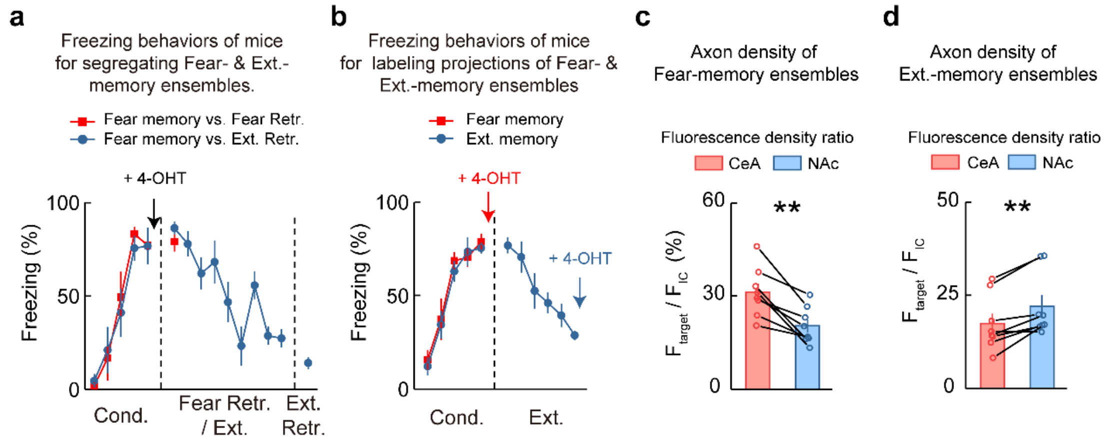

Supplementary Fig. 4. Characterization of segregation of fear- and extinction-memory ensembles in IC. a Freezing behaviors of FosTRAP2 mice for segregating fear and extinction memory ensembles, related to **Fig. 2a–d**. Freezing responses to the CS during fear conditioning (Cond.), fear retrieval (Fear Retr.), or extinction training (Ext.) and extinction retrieval (Ext. Retr.). While the freezing responses during conditioning were calculated by the freezing to every CS per trial, those during memory retrieval were calculated by the average freezing responses during every four trials. Fear Retr., n = 5 mice; Ext. Retr., n = 6 mice. Cond.: $F_{(1, 9)} = 0.04122$, $P = 0.8436$, two-way repeated measures ANOVA, main effects of groups. $t_{(9)} = 1.152$, $P = 0.2792$, Fear Retr. vs. the 1st block (four trials) of freezing during Ext. **b** Freezing behaviors of FosTRAP2 mice for identifying projections of IC fear and extinction memory ensembles, related to **Fig. 2e–i**. Freezing responses to the CS during fear conditioning (Cond.), fear retrieval (Fear Retr.), or extinction training (Ext.) and extinction retrieval (Ext. Retr.). While the freezing responses during conditioning were calculated by the freezing to every CS per trial, those during memory retrieval were calculated by the average freezing responses during every four trials. Fear memory, n = 8 mice; Ext. memory, n = 8 mice. Cond.: $F_{(1, 14)} = 0.2892$, $P = 0.5992$, two-way repeated measures ANOVA, main effects of groups. **c, d** Fluorescence densities of the axons from IC fear- and extinction-memory ensembles in NAc and CeA, related to **Fig. 2e–i**. Each group, n = 8 mice. **c** Fear memory, CeA vs. NAc: $t_{(7)} = 4.985$, $^{**}P = 0.0016$, two-tailed paired Student's *t*-test. **d** Ext. memory, CeA vs. NAc: $t_{(7)} = 4.2385$, $^{**}P = 0.0038$, two-tailed paired Student's *t*-test. Data are presented as mean values ± SEM and the error bar represents SEM. Source data are provided as a Source Data file.

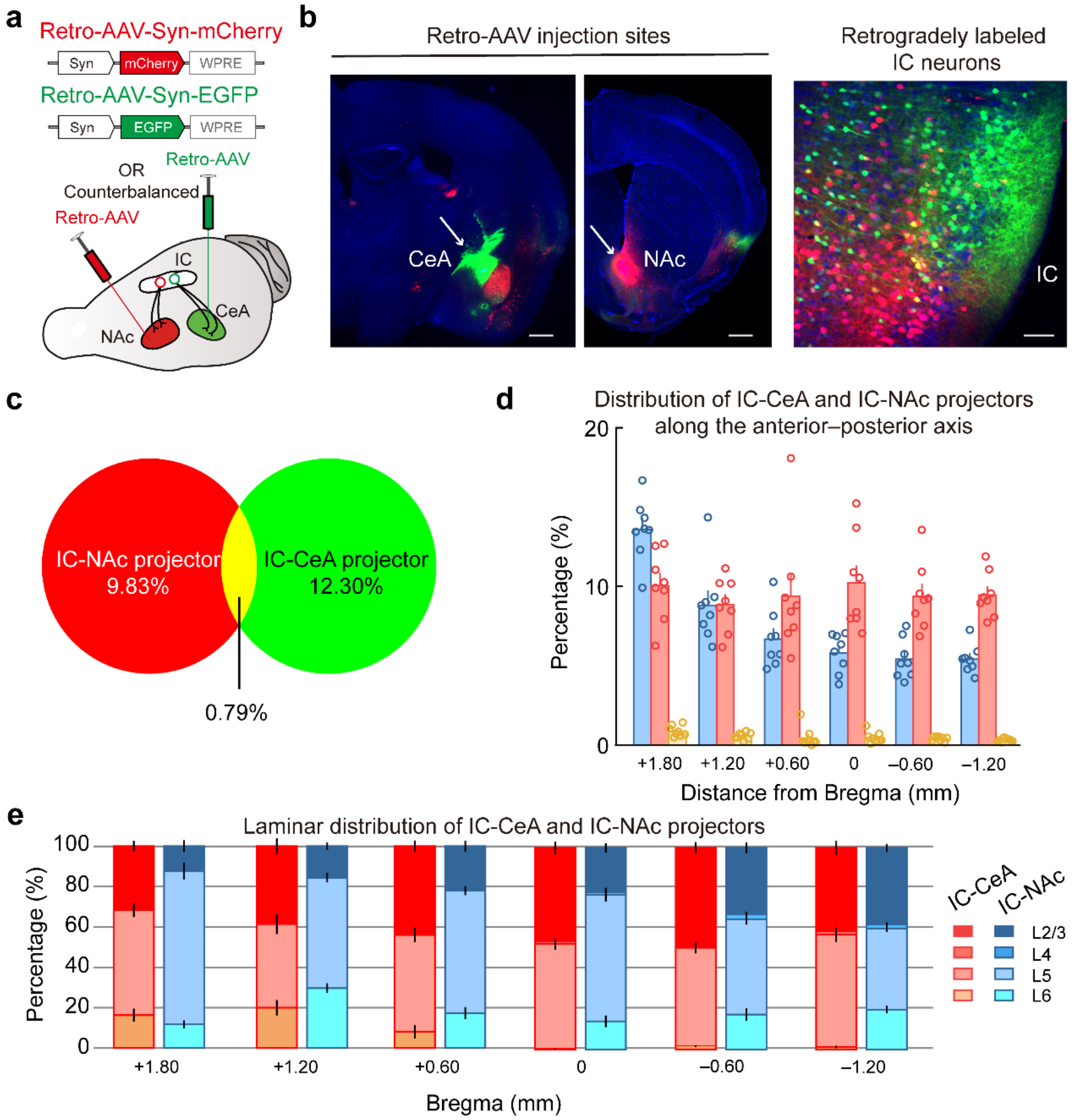

Supplementary Fig. 5. Largely non-overlapping IC neuronal subpopulations differentially project to the CeA and NAc. a Schematic representation of AAV injections to retrogradely label the IC projections to the CeA or NAc with Retro-AAV-Syn-EGFP or -mCherry, respectively, in a counterbalanced manner. **b** Representative images of AAV injection sites in the CeA (*left*, green), and NAc (*middle*, red), as well as retrograde labeling of IC neurons projecting to the CeA (termed as IC-CeA projectors, green) or that projecting to the NAc (termed as IC-NAc projectors, red), or both (yellow). Scale bars, 500 µm. **c** Overlapping neuronal populations of IC-CeA or IC-NAc projectors. Percentage of IC-CeA projectors or IC-NAc projectors in IC cells. n = 10 mice. **d** Distributions of IC-CeA and IC-NAc projectors along the anterior–posterior axis (+1.8 to −1.2 mm with respect to the bregma). **e**, Laminar distribution of IC-CeA and IC-NAc projectors along the anterior–posterior axis of the IC (+1.8 to −1.2 mm with respect to the bregma). **d−e** Percentage of input neurons in IC labeled from NAc or CeA. n = 8 mice. Data are presented as mean values ± SEM and the error bar

represents SEM. Source data are provided as a Source Data file.

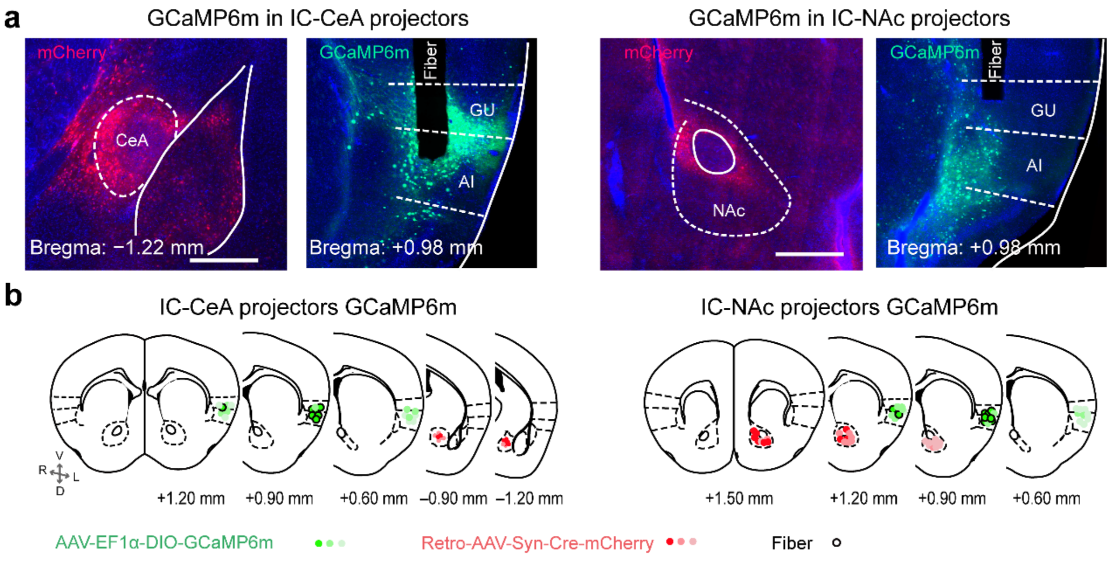

Supplementary Fig. 6. Histological verification of GCaMP6m expression and optical fiber placements for recording calcium response of IC-CeA (left) and IC-NAc (right) projectors, related to Fig. 4. a Representative images of Cre expression (mCherry) in a mouse that received Retro-AAV-Syn-Cre-mCherry injection into the CeA (left) or NAc (right) and AAV-EF1α-DIO-GCaMP6m injection into the IC. Scale bar, 500 μm. **b** Histological verification of GCaMP6m expression and optical fiber placements. IC-CeA group: n = 7 mice; IC-NAc group: n = 9 mice.

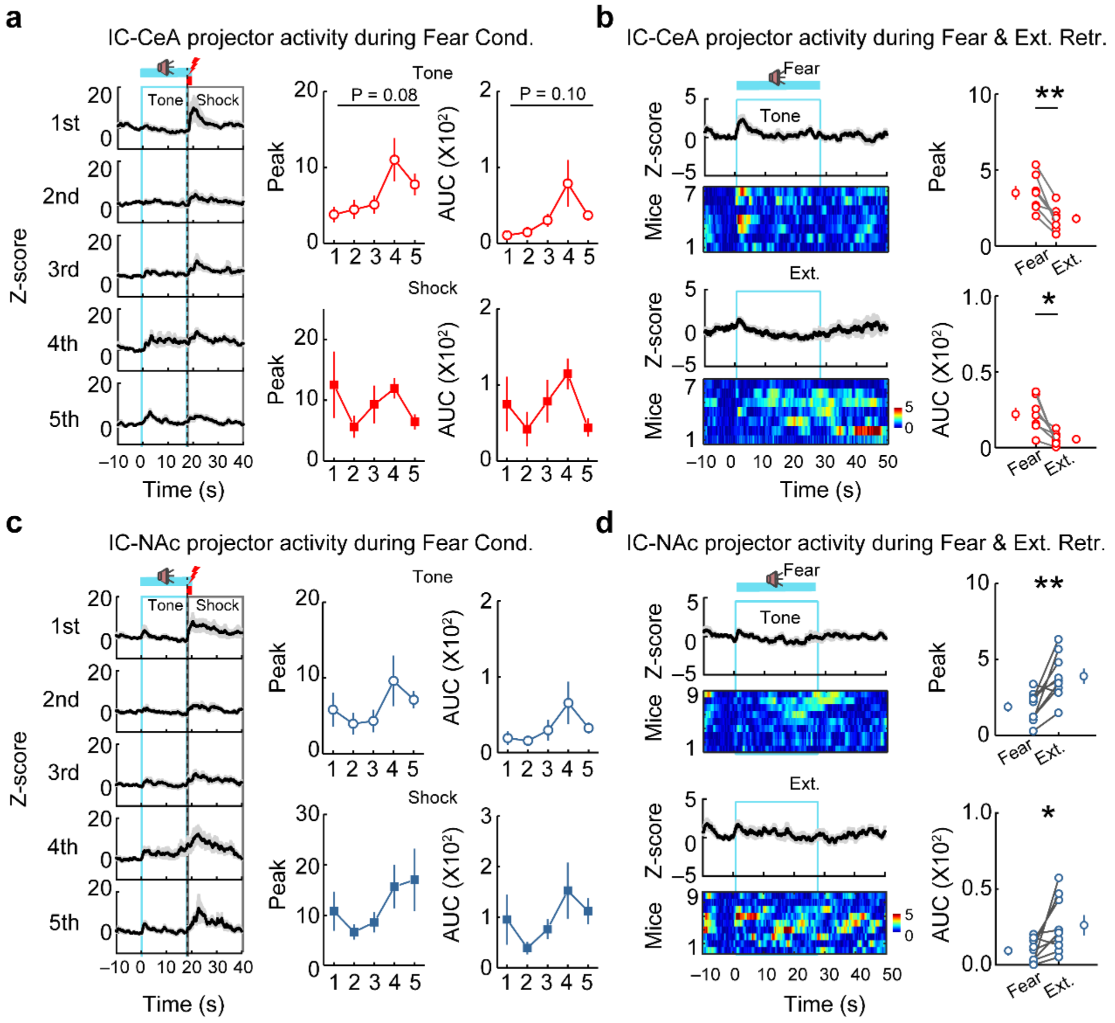

Supplementary Fig. 7. Z-score analysis of response patterns of IC-CeA and IC-NAc projectors to fear learning and fear- or extinction-memory retrieval. IC-CeA projectors, n = 7 mice; IC-NAc projectors, n = 9 mice. **a**, **b** Z-scores of calcium signals recorded from IC-CeA projectors. **a** Left: Average calcium signals (z-score) of IC-CeA projectors aligned to the onset of the CS during fear conditioning. Thick lines, mean; shaded areas, SEM. Right: the peak and the area under the curves (AUC) during tone (in cyan box) and shock (in gray box). Tone peak: $F_{(1.940, 11.64)} = 3.159$, $P = 0.0813$; tone AUC: $F_{(1.236, 7.414)} = 3.485$, $P = 0.0977$; shock peak: $F_{(1.679, 10.07)} = 1.278$, $P = 0.3125$; shock AUC: $F_{(2.055, 12.33)} = 1.970$, $P = 0.1804$, one-way repeated-measure ANOVA. **b** Average calcium signals (z-score) of IC-CeA projectors aligned to the onset of the CS during fear- or extinction-memory retrieval. Left upper: calcium signals during fear memory retrieval, average calcium signals and heatmap of calcium signals in each mouse. Left lower: similar as above for calcium signals during extinction memory retrieval. Right: The peak and AUC during tone in fear- and extinction-memory retrieval (in cyan box). Peak: $t_{(6)} = 5.051$, $^{*}P = 0.0023$; AUC: $t_{(6)} = 73.504$, $^{*}P = 0.0128$, two-tailed paired Student's *t*-test. **c**, **d** Similar to **a**, **b** for calcium signals recorded from IC-NAc projectors. **c** The responses of IC-NAc projectors to both tone and foot shock

kept constant during fear learning. Tone peak: $F_{(2.428, 19.42)} = 1.124$, $P = 0.3546$; tone AUC: $F_{(1.772, 14.17)} = 1.631$, $P = 0.2304$; shock peak: $F_{(2.335, 18.68)} = 1.244$, $P = 0.3160$; shock AUC: $F_{(1.944, 15.55)} = 1.237$, $P = 0.3161$, one-way repeated-measure ANOVA. **d** Peak: $t_{(8)} = 3.809$, $^{**}P = 0.0052$; AUC: $t_{(8)} = 2.626$, $^{*}P = 0.0304$, two-tailed paired Student's *t*-test. Data are presented as mean values ± SEM and the error bar represents SEM. Source data are provided as a Source Data file.

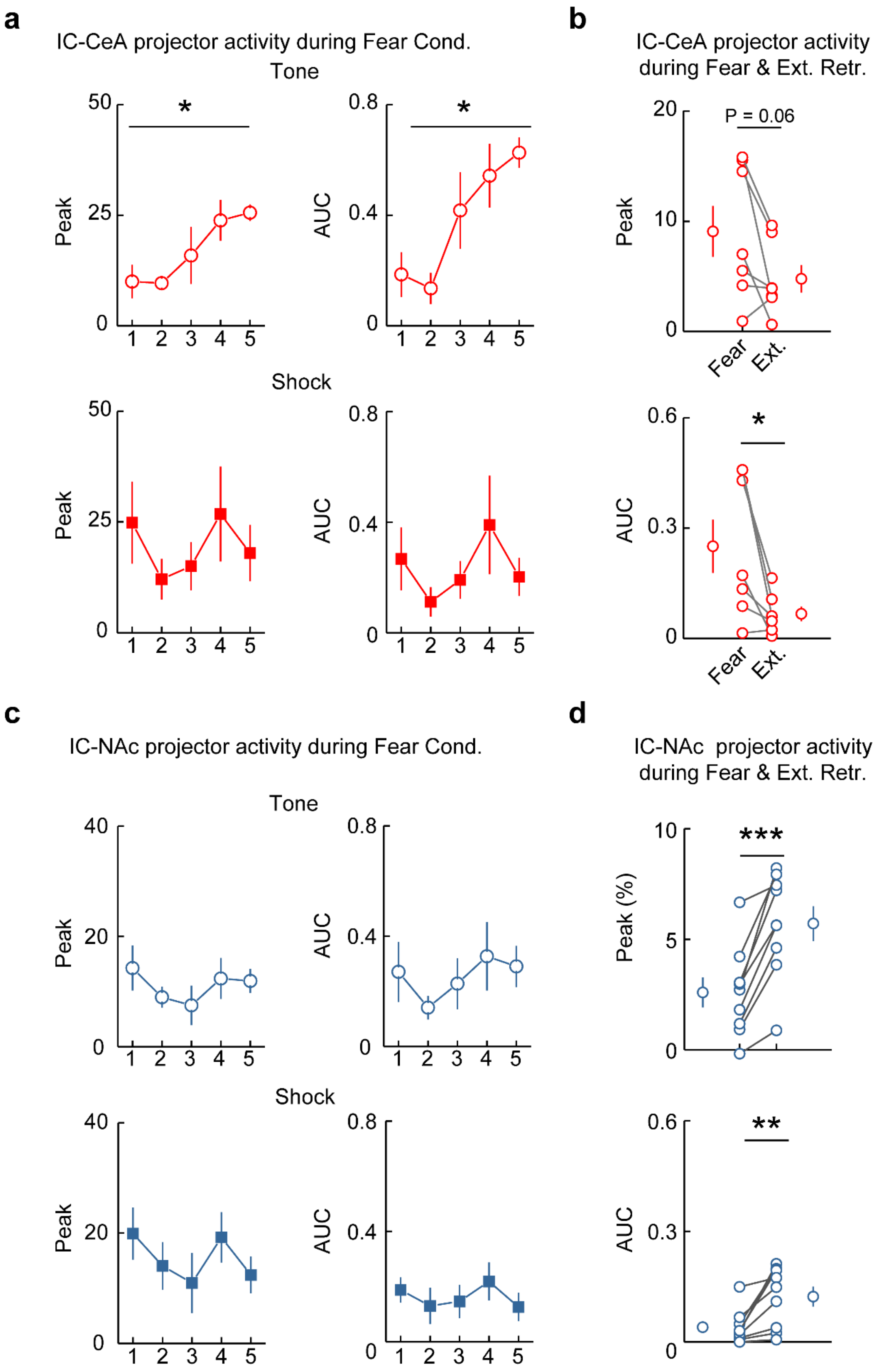

Supplementary Fig. 8. Quantification of response patterns of IC-CeA and IC-NAc

projectors to fear learning and fear- or extinction-memory retrieval by analyzing the initial responses to tone or shock. Only the first 5-s period of the CS and the 2-s period of the US were analyzed. IC-CeA projectors, n = 7 mice; IC-NAc projectors, n = 7 mice. **a**, **b** Calcium signals (ΔF/F) recorded from IC-CeA projectors. **a** The peak and the area under the curves (AUC) during tone (upper) and shock (lower). Tone peak: $F_{(2.353, 14.12)} = 3.822$, $^{*}P = 0.0417$; tone AUC: $F_{(2.317, 13.90)} = 5.837$, $^{*}P = 0.0120$; shock peak: $F_{(1.182, 7.095)} = 0.8557$, $P = 0.4050$; shock AUC: $F_{(1.260, 7.559)} = 1.087$, $P = 0.3490$, one-way repeated-measure ANOVA. **b** Calcium signals during fear and extinction memory retrieval. Peak: $t_{(6)} = 2.365$, $P = 0.0559$; AUC: $t_{(6)} = 3.069$, $^{*}P = 0.022$, two-tailed paired Student's *t*-test. **c**, **d** Similar to **a**, **b** for calcium signals recorded from IC-NAc projectors. **c** Responses of IC-NAc projectors to both tone and foot shock kept constant during fear learning. Tone peak: $F_{(1.951, 15.61)} = 0.6899$, $P = 0.5129$; tone AUC: $F_{(2.108, 16.86)} = 0.6266$, $P = 0.5544$; shock peak: $F_{(2.213, 17.71)} = 1.207$, $P = 0.3266$; shock AUC: $F_{(2.065, 16.52)} = 0.6326$, $P = 0.5485$, one-way repeated-measure ANOVA. **d** Activity of IC-NAc projectors during fear and extinction memory retrieval. Peak: $t_{(8)} = 6.285$, $^{***}P = 0.0002$; AUC: $t_{(8)} = 3.604$, $^{**}P = 0.0069$, two-tailed paired Student's *t*-test. Data are presented as mean values ± SEM and the error bar represents SEM. Source data are provided as a Source Data file.

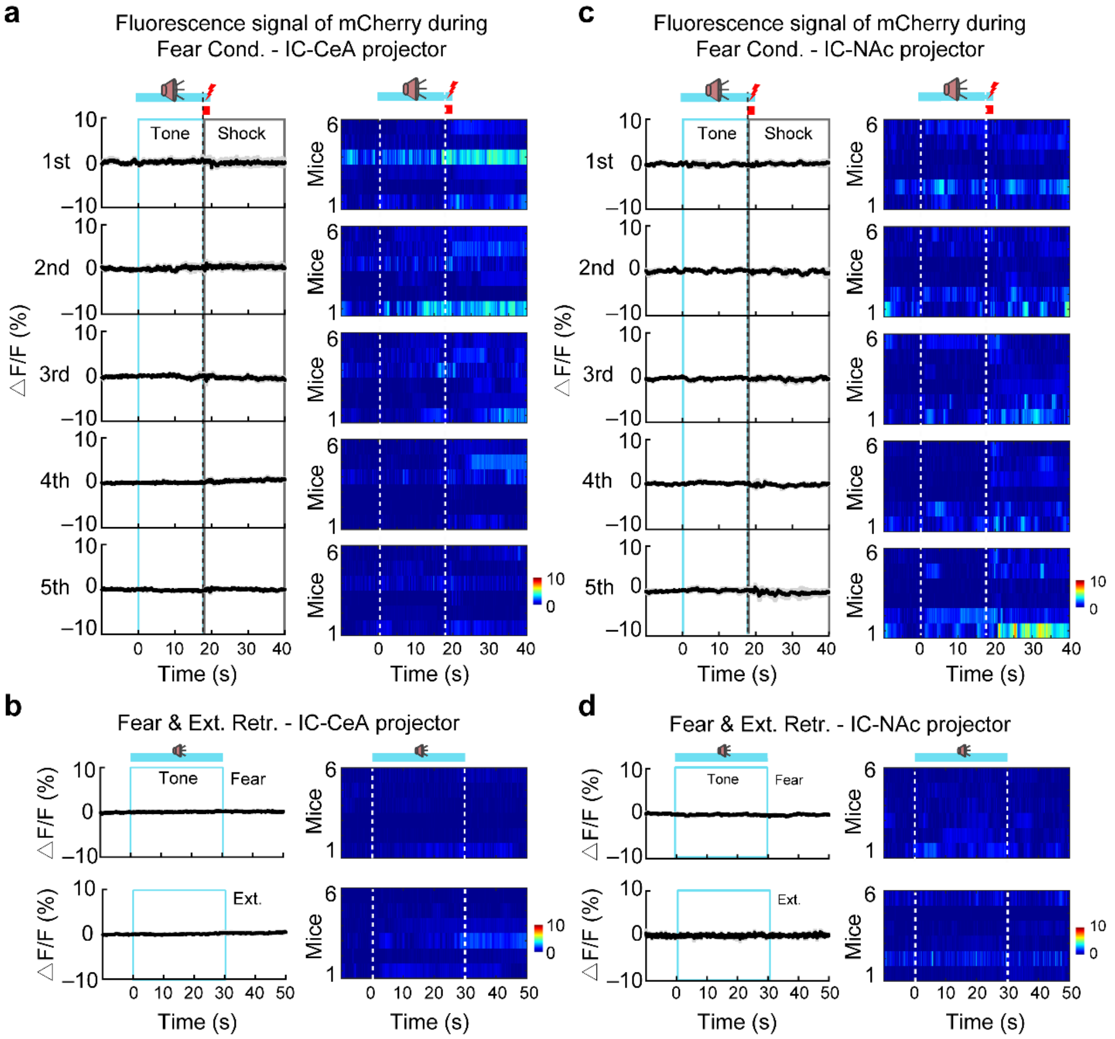

Supplementary Fig. 9. Lack of fluorescence change in IC-CeA and IC-NAc projectors expressing mCherry to fear learning and fear- or extinction-memory retrieval. a, **b** mCherry fluorescence intensity fluctuations (ΔF/F) recorded from IC-CeA projectors. **a** Left: Average fluorescence intensity fluctuations of IC-CeA projectors aligned to the onset of the CS during fear conditioning. Thick lines, mean; shaded areas, SEM. Right: heatmap illustration of fluorescence signals in each mouse. **b** Average fluorescence intensity fluctuations of IC-CeA projectors aligned to the onset of the CS during fear- or extinction-memory retrieval. Left: average fluorescence intensity fluctuations during fear (upper) or extinction (lower) memory retrieval. Right: heatmap illustration of fluorescence signals in each mouse. **c**, **d** Similar to **a**, **b** for the fluorescence signals recorded from IC-NAc projectors. Data are presented as mean values ± SEM and the error bar represents SEM.

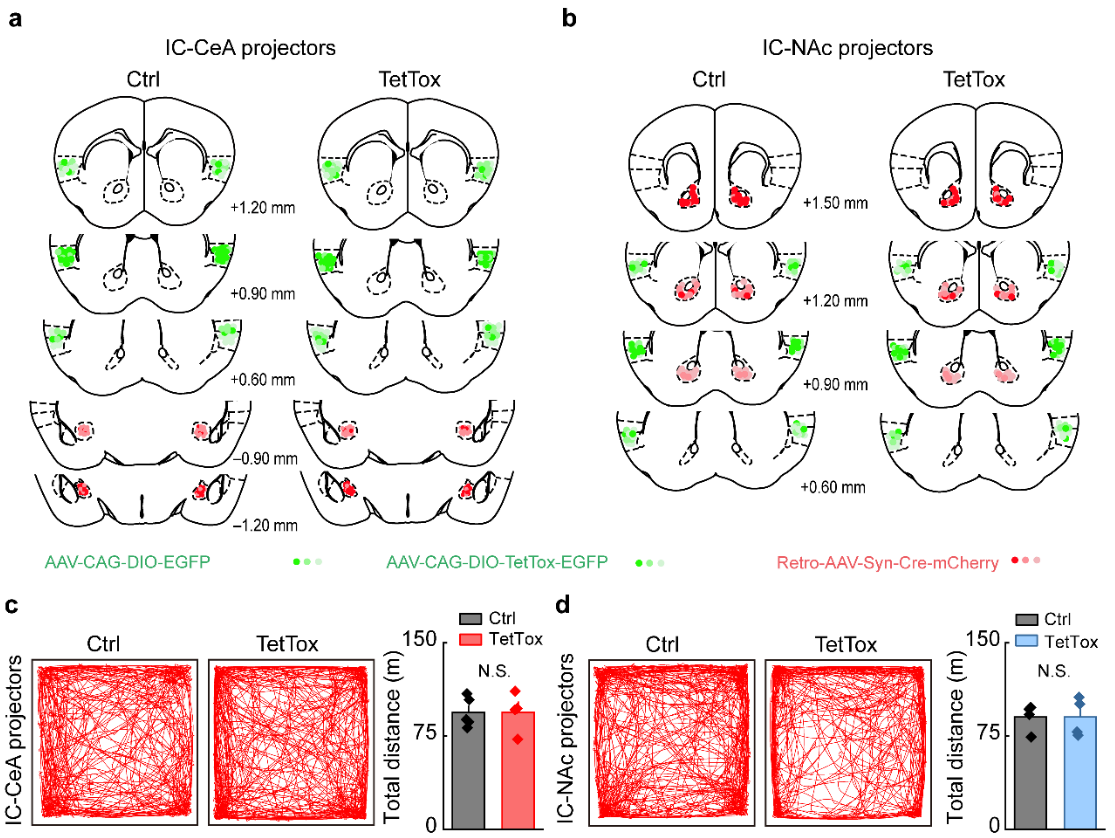

Supplementary Fig. 10. No effect of genetic silencing of IC-CeA or IC-NAc projectors on locomotor activity, related to Fig. 5a–d. a, **b** Histological confirmation of viral expression for genetic silencing of IC-CeA (**a**) and IC-NAc (**b**) projectors, related to **Fig. 5a–d**. **a** Ctrl, n = 15 mice; TetTox, n = 16 mice. **b** Ctrl, n = 10 mice; TetTox, n = 10 mice. **c**, **d** No effect of genetic silencing of IC-CeA (**c**) and IC-NAc (**d**) projectors on locomotor activity. Left, example traces of open field test for mice that received Retro-AAV-Syn-Cre-mCherry injection into the CeA (**c**) or NAc (**d**) and AAV-DIO-TetTox-EGFP (or -EGFP as Ctrl) injection into the IC. Right, Total distances travelled in the open field test. **c** IC-CeA projectors: Ctrl, n = 5 mice; TetTox, n = 4 mice. $t_{(7)}$ = 0.03261, P = 0.9749, two-tailed unpaired Student's *t*-test. **d** IC-NAc projectors: Ctrl, n = 4 mice; TetTox, n = 4 mice. $t_{(6)}$ = 0.005320, P = 0.9959, two-tailed unpaired Student's *t*-test. Data are presented as mean values ± SEM and the error bar represents SEM. Source data are provided as a Source Data file.

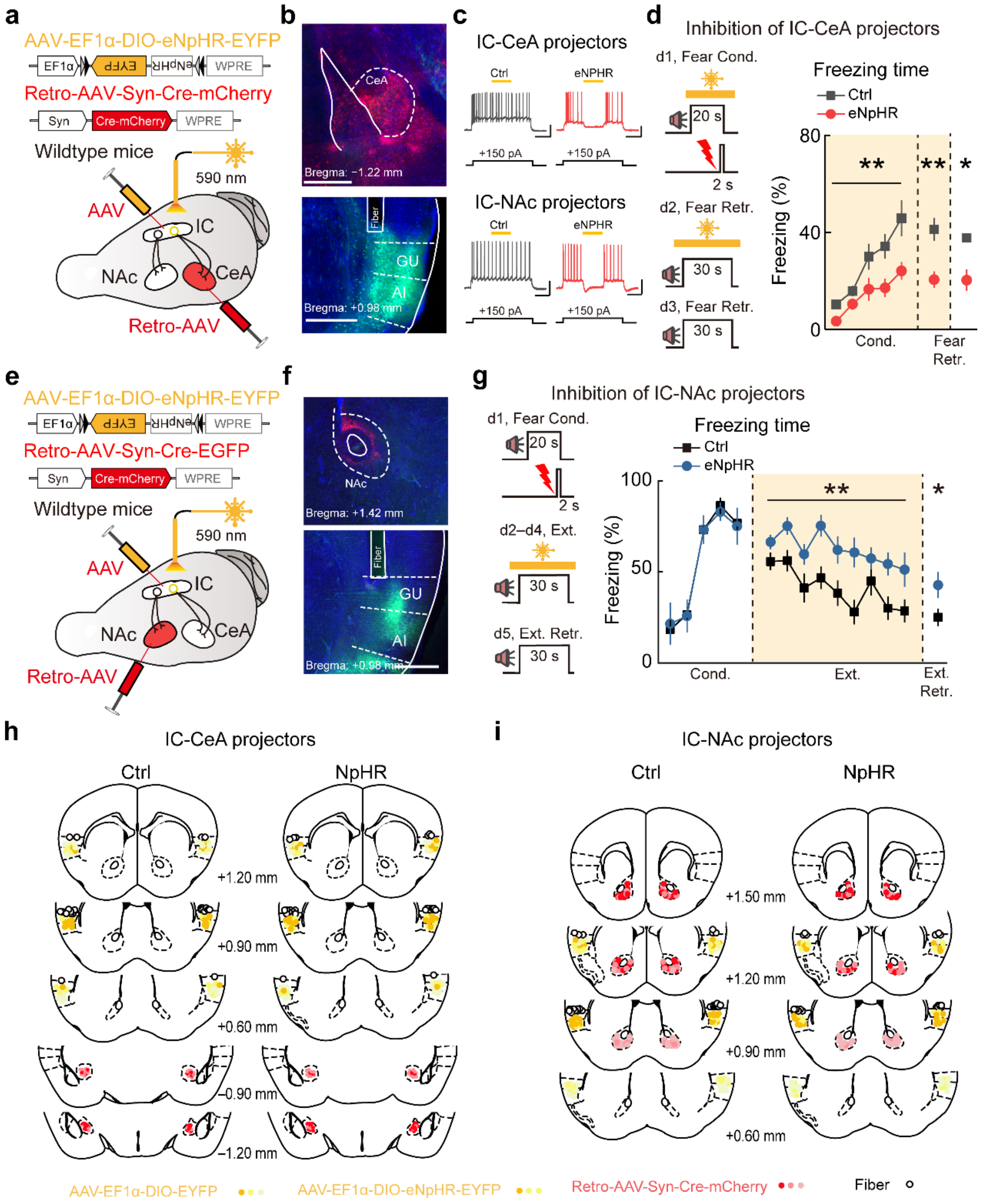

Supplementary Fig. 11. Effects of optogenetic inhibition of IC-CeA or IC-NAc projectors on fear and extinction memories, respectively. a–d Effects of optogenetic inhibition of IC-CeA projectors on fear conditioning and memory retrieval. **a**, **e** Schematic of AAV injections. **b**, **f** Representative images. Scale bar, 500 µm. **c** Electrophysiological verification of eNpHR-EYFP expression in IC projectors. Scale bars, 500 ms, 40 mV. **d**, **g** Effects of optogenetic inhibition of IC-CeA (**d**) or IC-NAc (**g**) projectors. Left: behavioral protocols. Right: behavioral results. The yellow-shadowed box denotes the light-on phase. While the freezing responses during conditioning were calculated by the freezing to every CS per trial, those during memory

retrieval were calculated by the average freezing responses during every four trials. **d** Ctrl (EYFP), n = 11 mice; eNpHR, n = 11 mice. Cond.: $F_{(1, 20)} = 8.393$, $^{**}P = 0.0089$, two-way repeated measures ANOVA; Fear Retr. with light, $t_{(20)} = 3.581$, $^{**}P = 0.0019$, two-tailed unpaired Student's *t*-test; Fear Retr. without light, $t_{(20)} = 2.374$, $^{*}P = 0.0277$, two-tailed unpaired Student's *t*-test. **g** Ctrl (EYFP), n = 12 mice; eNpHR, n = 9 mice. Cond.: $F_{(1, 19)} = 0.0081$, $P = 0.9291$, two-way repeated-measures ANOVA; Ext.: $F_{(1, 19)} = 11.11$, $^{**}P = 0.0035$, two-way repeated-measures ANOVA; Retr.: $t_{(19)} = 2.289$, $^{*}P = 0.0337$, two-tailed unpaired Student's *t*-test. **h**, **i** Histological confirmation of optical fiber placements for optogenetic inhibition of IC-CeA (**h**) or IC-NAc (**i**) projectors. **h** IC-CeA projectors, Ctrl, n = 11 mice; eNpHR, n = 11 mice. **i** IC-NAc projectors, Ctrl, n = 12 mice; eNpHR, n = 9 mice. Data are presented as mean values ± SEM and the error bar represents SEM. Source data are provided as a Source Data file.

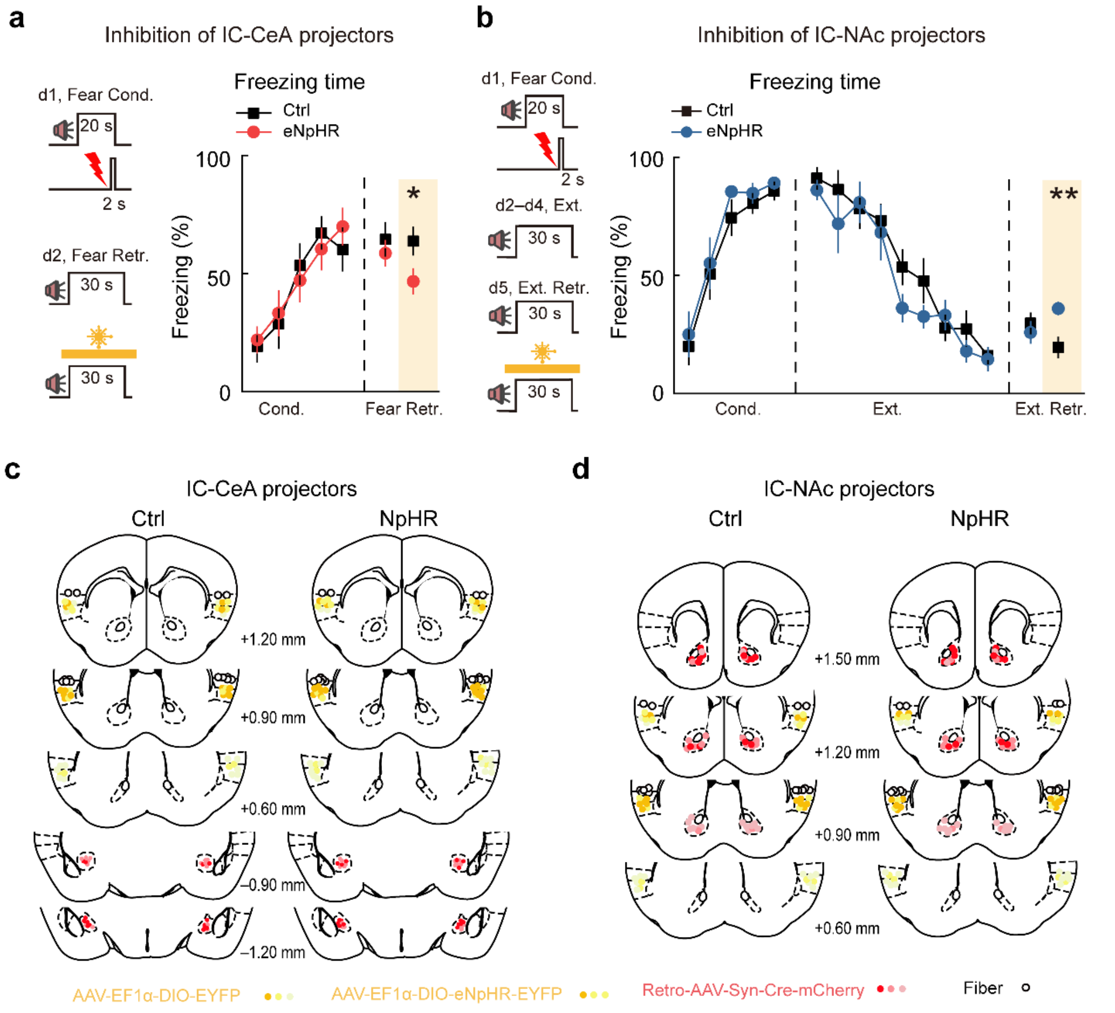

Supplementary Fig. 12. Effects of optogenetic inhibition of IC-CeA or IC-NAc projectors on fear and extinction memory retrieval, respectively. a Effects of optogenetic inhibition of IC-CeA projectors on fear memory retrieval. Left: behavioral protocols. Right: behavioral responses. The yellow-shadowed box denotes the light-on phase. Freezing responses to the CS during fear conditioning and memory retrieval with or without yellow light. While the freezing responses during conditioning were calculated by the freezing to every CS per trial, those during memory retrieval were calculated by the average freezing responses during every four trials. Ctrl (EYFP), n = 7 mice; eNpHR, n = 8 mice. Cond.: $F_{(1,\ 13)} = 0.0089$, $P = 0.9263$, two-way repeated measures ANOVA; Fear Retr. without light, $t_{(13)} = 0.6806$, $P = 0.5081$, two-tailed unpaired Student's *t*-test; Fear Retr. with light, $t_{(13)} = 2.193$, $^{*}P = 0.0471$, two-tailed unpaired Student's *t*-test. **b** Effects of optogenetic inhibition of IC-NAc projectors on extinction memory retrieval. Left: behavioral protocols. Right: behavioral responses. The yellow-shadowed box denotes the light-on phase. Freezing responses to the CS during fear conditioning and memory retrieval with or without yellow light. While the freezing responses during conditioning were calculated by the freezing to every CS per trial, those during memory retrieval were calculated by the average freezing responses

during every four trials. Ctrl (EYFP), n = 7 mice; eNpHR, n = 7 mice. Cond.: $F_{(1, 12)}$ = 0.8949, P = 0.3628; Ext.: $F_{(1, 12)}$ = 1.118, P = 0.3112, two-way repeated measures ANOVA. Ext. Retr. without light, $t_{(12)}$ = 0.6217, P = 0.5458, two-tailed unpaired Student's *t*-test; Ext. Retr. with light, $t_{(12)}$ = 3.351, $^{**}P$ = 0.0058, two-tailed unpaired Student's *t*-test. **c**, **d** Histological confirmation of optical fiber placements for optogenetic inhibition of IC-CeA (**c**) or IC-NAc (**d**) projectors. **c** IC-CeA projectors, Ctrl, n = 7 mice; eNpHR, n = 8 mice. **d** IC-NAc projectors, Ctrl, n = 7 mice; eNpHR, n = 7 mice. Data are presented as mean values ± SEM and the error bar represents SEM. Source data are provided as a Source Data file.

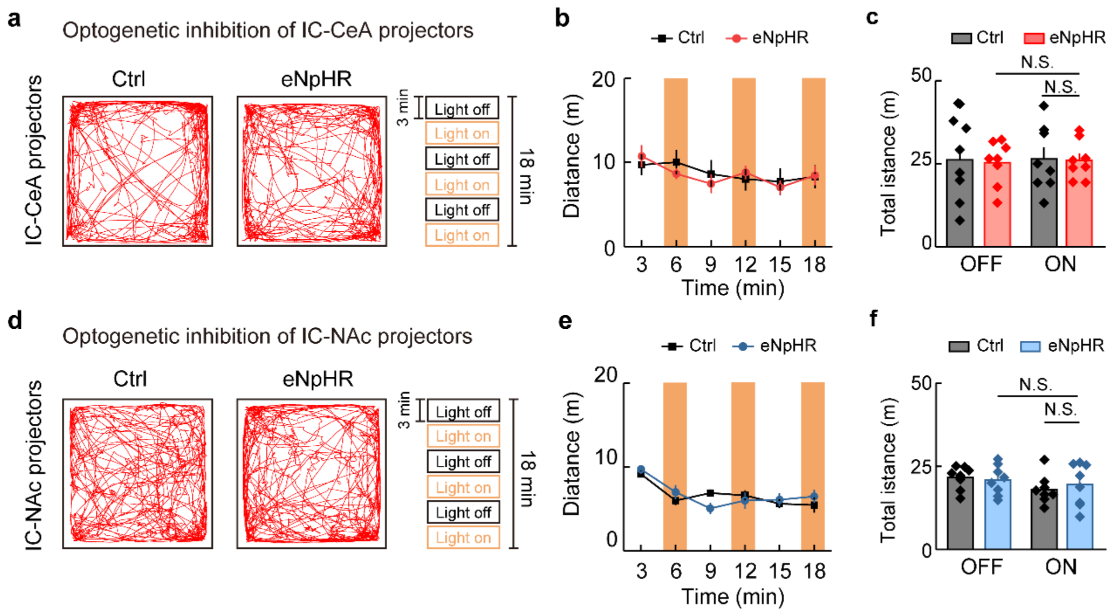

Supplementary Fig. 13. No effect of optogenetic inhibition of IC-CeA (a–c) or IC-NAc (d–f) projectors on locomotor activity. a, **d** Left: Example traces of open field test for mice that received Retro-AAV-Syn-Cre-mCherry injection into the CeA (**a**) or NAc (**b**) and AAV-DIO-eNpHR-EYFP (or -EYFP as Ctrl) injection into the IC. Right: behavioral protocols. **b**, **e** Effect of optogenetic inhibition of IC-CeA (**b**) or IC-NAc (**e**) projectors on locomotor activity in the open filed test. Each point represents the distance traveled in the open field in a 3-min light on (orange box) or light-off period. **b** IC-CeA projectors, Ctrl, n = 8 mice; eNpHR, n = 8 mice. $F_{(1, 14)} = 0.02099$, $P = 0.8869$. **e** IC-NAc projectors, Ctrl, n = 8 mice; eNpHR, n = 8 mice. $F_{(1, 14)} = 0.02189$, $P = 0.8845$. **c**, **f** Total distances travelled in the open field for both light-on and light-off periods. **c** IC-CeA projectors, light on, Ctrl vs. eNpHR, $t_{(14)} = 0.1061$, $P = 0.9170$, two-tailed unpaired Student's *t*-test; eNpHR: light off vs. light on, $t_{(7)} = 0.3404$, $P = 0.7435$, two-tailed paired Student's *t*-test. **f** IC-NAc projectors, light on, Ctrl vs. eNpHR, $t_{(14)} = 0.5240$, $P = 0.6085$, two-tailed unpaired Student's *t*-test; eNpHR: light off vs. light on, $t_{(7)} = 0.9368$, $P = 0.38$, two-tailed paired Student's *t*-test. Data are presented as mean values ± SEM and the error bar represents SEM. Source data are provided as a Source Data file.

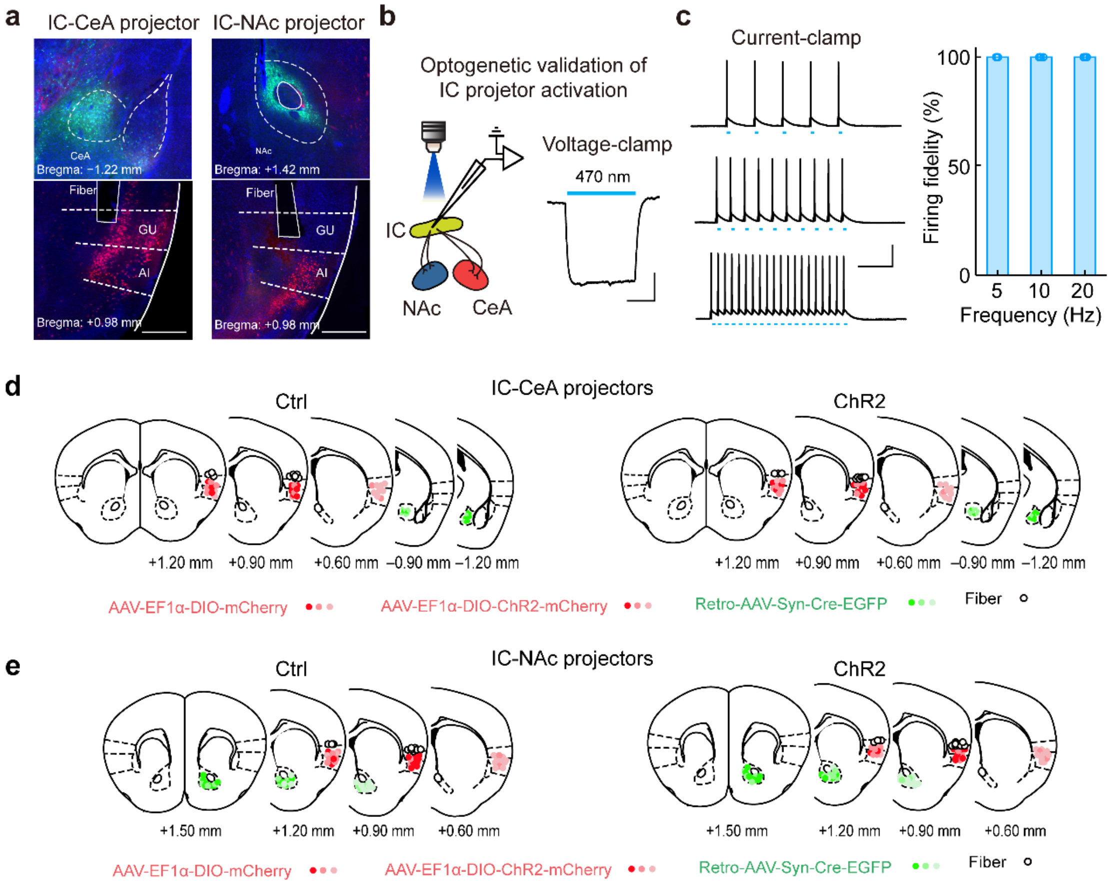

Supplementary Fig. 14. Histological and electrophysiological verifications of optogenetic activation of IC-CeA or IC-NAc projectors. a Representative images of Cre expression (EGFP) in a mouse that received Retro-AAV-Syn-Cre-EGFP injection into the CeA (*Left*) or NAc (*Right*) and AAV-EF1α-DIO-ChR2-mCherry (or -mCherry) injection into the IC. Scale bar, 500 μm. **b** Left: Experimental schemes. Right: ChR2-mediated currents were evoked by 500-ms pulses of light (470 nm, cyan horizontal bar) at –70 mV in voltage-clamp mode. Scale bars, 200 ms, 50 pA. **c** Left: Action potentials (APs) were evoked in IC projectors in current-clamp mode by 1-ms pulses of photostimuli at different frequencies (cyan dots). Scale bars, 250 ms, 40 mV. Right: Summary plot of AP firing fidelity versus photostimulation frequency. n = 5. **d**, **e** Histological confirmation of optical fiber placements for optogenetic activation of IC-CeA (**d**) or IC-NAc (**e**) projectors, related to **Fig. 4e–g**. **d** IC-CeA projectors, Ctrl, n = 9 mice; ChR2, n = 9 mice. **e** IC-NAc projectors, Ctrl, n = 11 mice; ChR2, n = 11 mice.

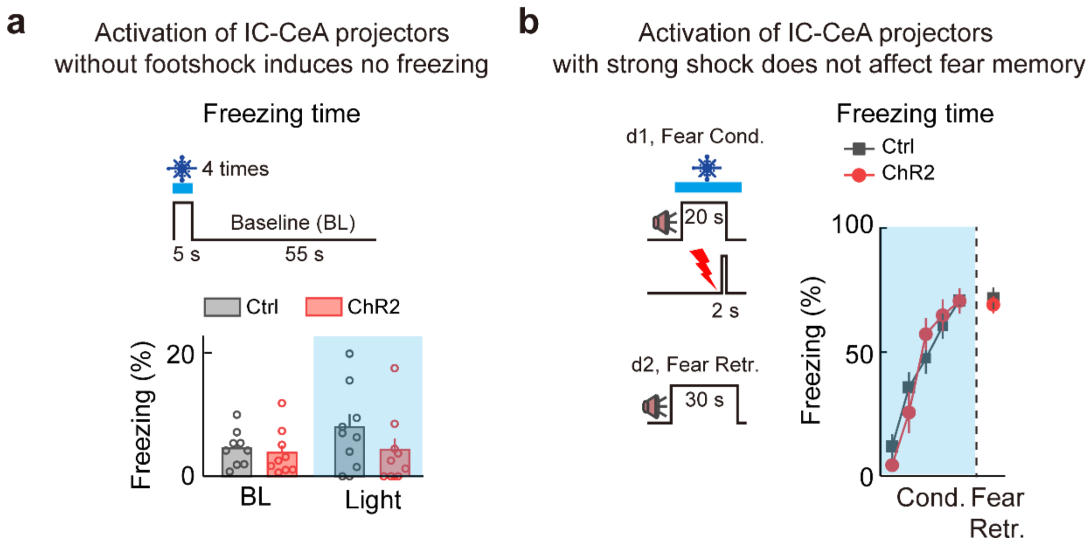

Supplementary Fig. 15. Effects of optogenetic activation of IC-CeA projectors on unconditioned fear behaviors or conditioned fear behaviors induced by strong conditioning protocols. a, Effects of optogenetic activation of IC-CeA projectors on unconditioned fear behaviors. Upper: behavioral protocols. Lower: effects of light activation of IC-CeA projectors on unconditioned freezing behaviors. The blue-shadowed boxes denote the light-on phase. Freezing responses to the context only (baseline, BL) or to the light. Ctrl (mCherry), n = 9 mice, ChR2, n = 9 mice. BL: $t_{(16)} = 0.4516$, $P = 0.6576$; Light: $t_{(16)} = 1.3118$, $P = 0.2081$, two-tailed unpaired Student's *t*-test. **b**, Effects of optogenetic activation of IC-CeA projectors on strong conditioning and subsequent memory retrieval. Left: behavioral protocols. Right: behavioral data. The blue-shadowed boxes denote the light-on phase. While the freezing responses during fear conditioning were calculated by the freezing to every CS per trial, those during memory retrieval were calculated by the average freezing responses during every four trials. Ctrl (mCherry), n = 10 mice, ChR2, n = 10 mice. Cond.: $F_{(1, 18)} = 0.01683$, $P = 0.8982$, two-way repeated measures ANOVA; Fear Retr.: $t_{(18)} = 0.4640$, $P = 0.6482$, two-tailed unpaired Student's *t*-test. Data are presented as mean values ± SEM and the error bar represents SEM. Source data are provided as a Source Data file.

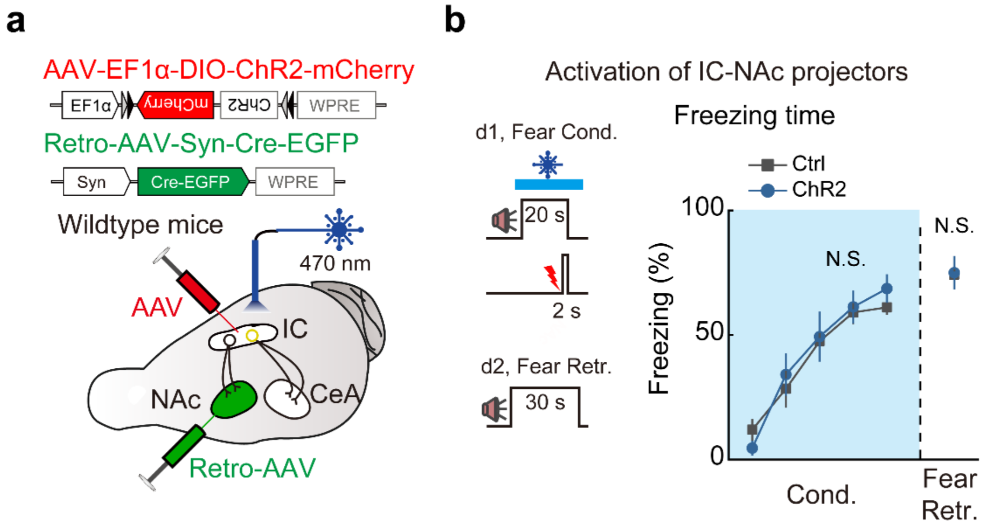

Supplementary Fig. 16. Effects of optogenetic activation of IC-NAc projectors on fear conditioning and subsequent memory retrieval. a Schematic of AAV injections for expressing ChR2 in IC-NAc projectors. **b** Left: behavioral protocols. Right: behavioral data. The blue-shadowed boxes denote the light-on phase. While the freezing responses during fear conditioning were calculated by the freezing to every CS per trial, those during memory retrieval were calculated by the average freezing responses during every four trials. Ctrl (mCherry), n = 8 mice, ChR2, n = 8 mice. Cond.: $F_{(1, 18)} = 0.1087$, $P = 0.7466$, two-way repeated measures ANOVA; Fear Retr.: $t_{(18)} = 0.09319$, $P = 0.9271$, two-tailed unpaired Student's *t*-test. N.S., no significant difference. Data are presented as mean values ± SEM and the error bar represents SEM. Source data are provided as a Source Data file.

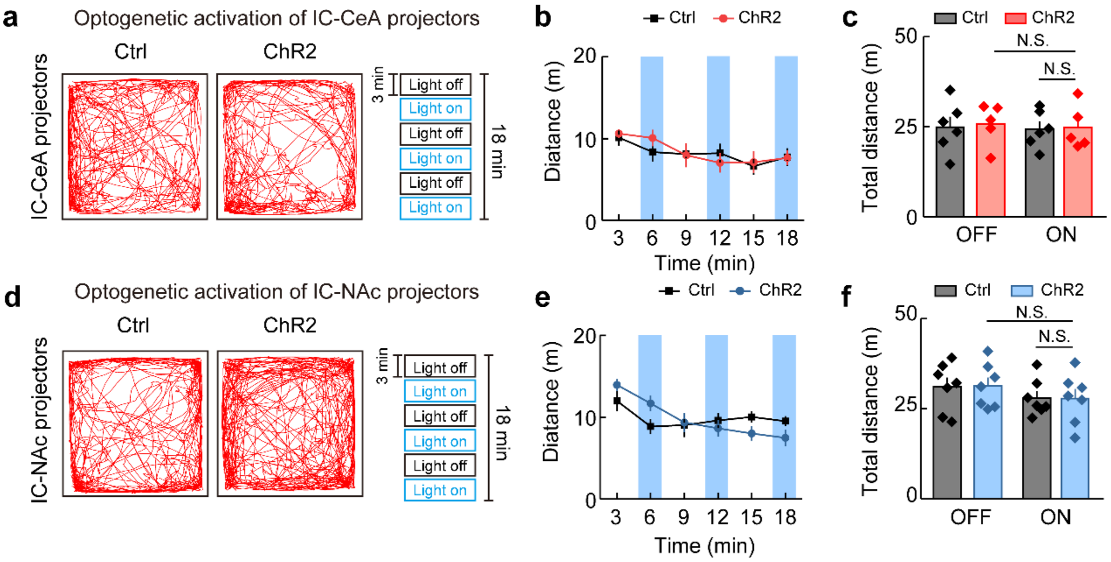

Supplementary Fig. 17. No effect of optogenetic activation of IC-CeA (a–c) or IC-NAc (d–f) projectors on locomotor activity. **a**, **d** Left: Example traces of open field test for mice that received Retro-AAV-Syn-Cre-EGFP injection into the CeA (**a**) or NAc (**d**) and AAV-DIO-ChR2-mCherry (or -mCherry as Ctrl) injection into the IC. Right: behavioral protocols. **b**, **e** Effect of optogenetic activation of IC-CeA (**b**) or IC-NAc (**e**) projectors on locomotor activity in the open filed test. Each point represents the distance traveled in the open field in a 3-min light on (blue box) or light-off period. **b** IC-CeA projectors, Ctrl, n = 6 mice; ChR2, n = 5 mice. $F_{(1, 9)} = 0.03389$, $P = 0.\ 8580$, two-way repeated measures ANOVA. **e** IC-NAc projectors, Ctrl, n = 7 mice; ChR2, n = 7 mice. $F_{(1, 12)} = 0.0003$, $P = 0.9873$, two-way repeated measures ANOVA.. **c**, **f** Total distances travelled in the open field for both light-on and light-off periods. **c** IC-CeA projectors, light on, Ctrl vs. ChR2, $t_{(9)} = 0.1291$, $P = 0.9001$, two-tailed unpaired Student's *t*-test; ChR2: light off vs. light on, $t_{(4)} = 0.4178$, $P = 0.6975$, two-tailed paired Student's *t*-test. **f** IC-NAc projectors, light on, Ctrl vs. ChR2, $t_{(12)} = 0.05129$, $P = 0.9599$, two-tailed unpaired Student's *t*-test; ChR2: light off vs. light on, $t_{(6)} = 2.407$, $P = 0.0528$, two-tailed paired Student's *t*-test. Data are presented as mean values ± SEM and the error bar represents SEM. Source data are provided as a Source Data file.

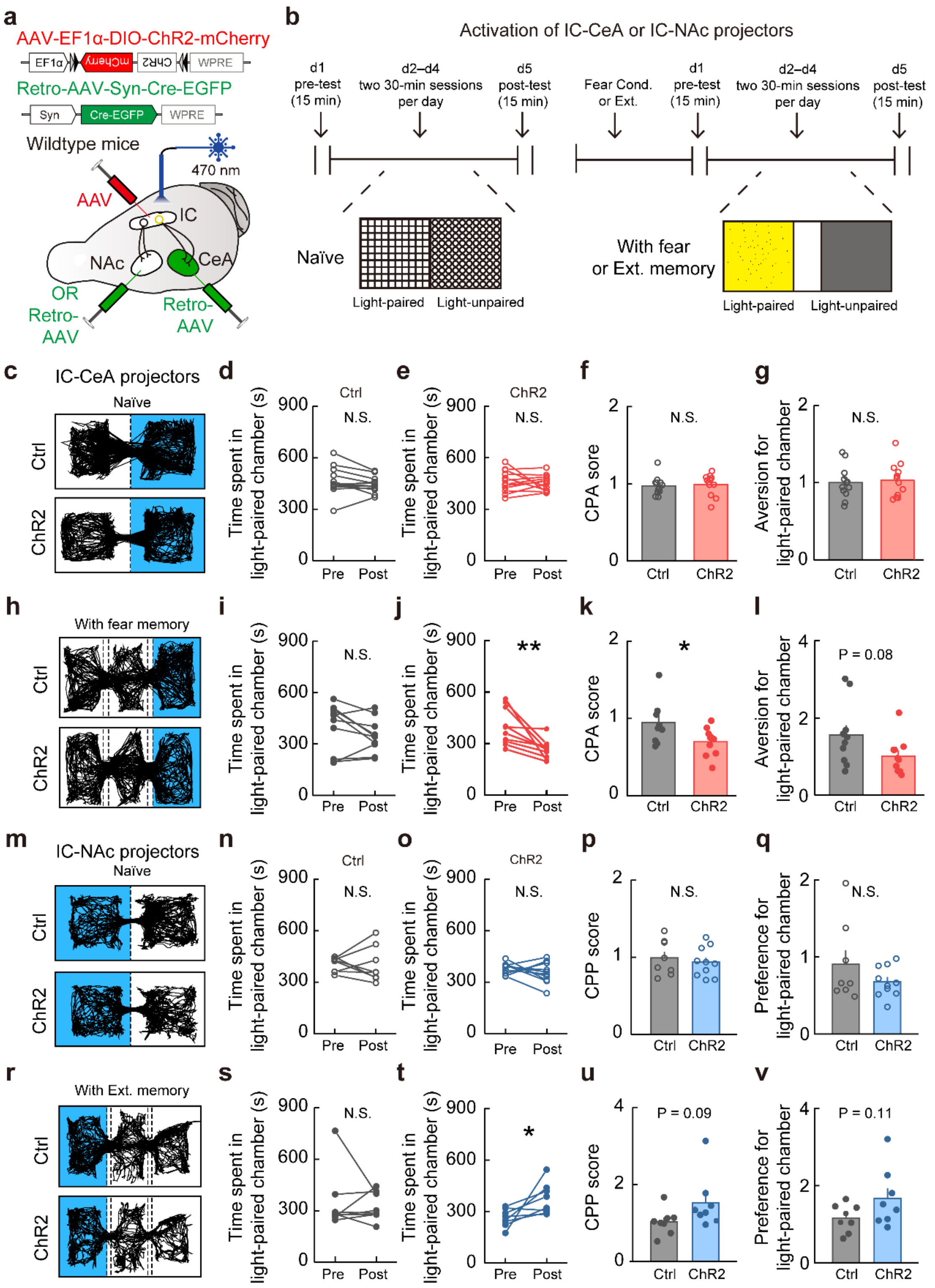

Supplementary Fig. 18. IC-CeA or IC-NAc projectors contribute to fear- or extinction-memory-associated conditioned place aversion or preference, respectively. a Schematic of AAV injections. **b** Experimental designs. **c–e**, **h-j** Representative paths (**c**, **h**) and individual data of the durations of time spent in the light-paired chamber (**d**, **e**, **i**, and **j**) before (Naïve, **c–e**) and after (Experienced, **h–j**)

fear conditioning. **f**, **g**, **k**, **l** Quantification of fear memory-associated CPA. Naïve, mCherry, n = 12 mice; ChR2: n = 12 mice; with fear memory, mCherry, n = 10 mice; ChR2, n = 10 mice. **d** $t_{(11)} = 1.384$, $P = 0.1939$; **e** $t_{(11)} = 0.5440$, $P = 0.5973$, two-tailed paired Student's *t* test; **f** $t_{(22)} = 0.3022$, $P = 0.7653$; **g** $t_{(22)} = 0.3535$, $P = 0.7271$, two-tailed unpaired Student's *t* test; **i** $t_{(9)} = 1.571$, $P = 0.1506$; **j** $t_{(9)} = 3.730$, $P = 0.0047$, two-tailed paired Student's *t* test; **k** $t_{(18)} = 2.407$, $P = 0.027$; **l** $t_{(18)} = 1.822$, $P = 0.08521$, two-tailed unpaired Student's *t* test. **m**–**v** Representative paths (**m**, **p**) and individual data of the durations of time spent in the light-paired chamber (**n**, **o**, **q**, and **r**) before (Naïve, **m**–**o**) and after (Experienced, **p**–**r**) extinction training. **p**, **q**, **u**, **v** Quantification of extinction memory-associated CPP. Naïve, mCherry, n = 8 mice; ChR2: n = 10 mice; with extinction memory, mCherry, n = 8 mice; ChR2, n = 8 mice. **n** $t_{(7)} = 0.1074$, $P = 0.9175$; **o** $t_{(9)} = 1.156$, $P = 0.2776$, two-tailed paired Student's *t* test; **p** $t_{(16)} = 0.5478$, $P = 0.5914$; **q** $t_{(16)} = 1.292$, $P = 0.2149$, two-tailed unpaired Student's *t* test; **s** $t_{(7)} = 0.4227$, $P = 0.6852$; **t** $t_{(7)}=2.615$, $P = 0.0347$, paired Student's *t* test; **u** $t_{(14)} = 1.818$, $P = 0.0905$; **v** $t_{(14)} = 1.702$, $P = 0.1109$, two-tailed unpaired Student's *t* test. Data are presented as mean values ± SEM and the error bar represents SEM. Source data are provided as a Source Data file.

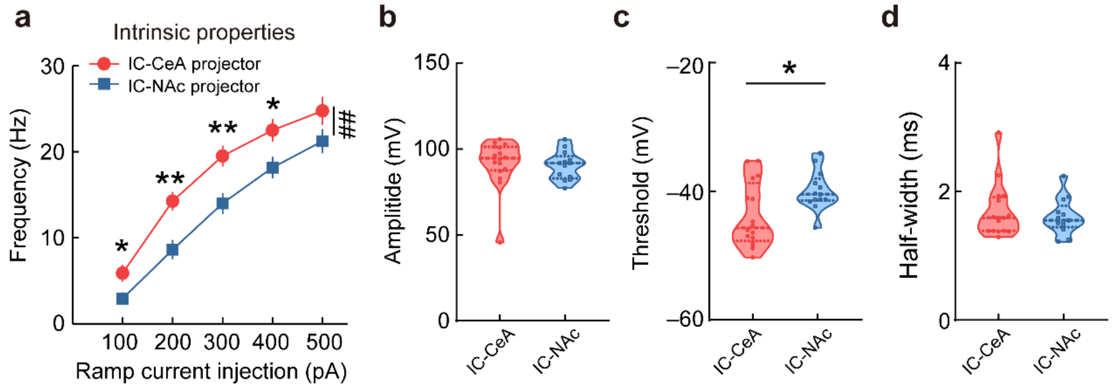

Supplementary Fig. 19. Quantification of electrophysiological properties of separate IC-CeA and IC-NAc projectors. a Frequencies of action-potential (AP) discharges evoked by various ramp- (0–100, 0–200, 0–300, 0–400, 0–500 pA, 500 ms) current intensities. IC-CeA projectors, n = 13 neurons of 7 mice; IC-NAc projectors, n = 16 neurons of 6 mice. All: $F_{(1, 27)} = 7.873$, $^{\#\#}P = 0.0092$, Two-way repeated-measures ANOVA. 100 pA: $t_{(27)} = 2.323$, $^{*}P = 0.0279$; 200 pA: $t_{(27)} = 3.571$, $^{**}P = 0.0014$; 300 pA: $t_{(27)} = 3.156$, $^{**}P = 0.0039$; 400 pA: $t_{(27)} = 2.391$, $^{*}P = 0.024$; 500 pA: $t_{(27)} = 1.591$, $P = 0.1232$, two-tailed unpaired Student's *t*-test. Data are presented as mean values ± SEM and the error bar represents SEM. **b**–**d** Summary data for AP amplitude (**b**), threshold (**c**), and half-width (**d**) obtained from slice recording data shown in (**a**) but also Fig. 5b. **b** amplitude: $t_{(27)} = 0.2680$, $P = 0.7907$; **c** threshold: $t_{(27)} = 2.432$, $^{*}P = 0.0219$; **d** half-width: $t_{(27)} = 0.6203$, $P = 0.5403$, two-tailed unpaired Student's *t*-test. Source data are provided as a Source Data file.

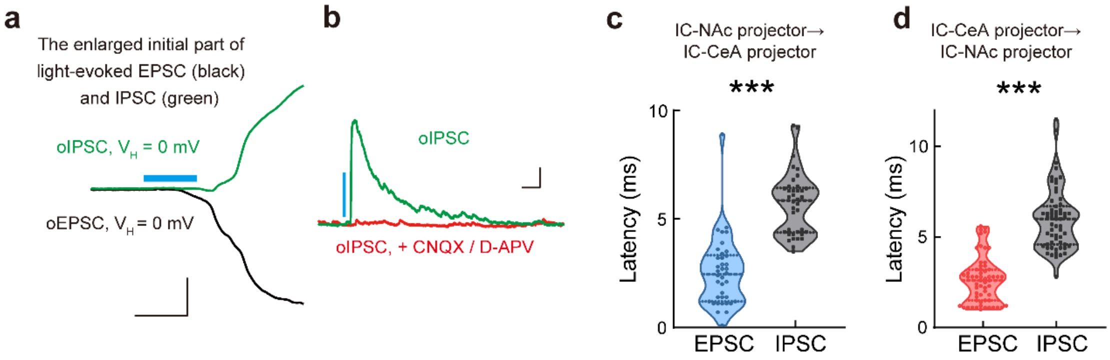

Supplementary Fig. 20. Electrophysiological characterization of synaptic projections from the IC-CeA projector to the IC-NAc projector and vice versa. a The expanded initial segments of light-evoked EPSC (black) and IPSC (green) in an example IC neuron. Blue bar, duration of light stimulation (5 ms). Scale bars, 5 ms, 500 pA. **b** oIPSC with (red) or without (green) glutamatergic AMPAR antagonist, CNQX (20 μM), and the NMDAR antagonist, D-APV (50 μM). The blue vertical bar above traces indicates the photostimulation. Scale bars, 50 ms, 200 pA. **c** Quantification of latency of synaptic connectivity on IC-NAc projectors driven by activation of IC-CeA projectors in the local circuits. n = 38 neurons of 15 mice. $t_{(37)} = 10.71$, $^{***}P = 1.1490E{-}13$, two-tailed paired Student's *t*-test. **d** Quantification of latency of synaptic connectivity on IC-CeA projectors driven by activation of IC-NAc projectors in the local circuits. n = 59 neurons of 22 mice. $t_{(58)} = 14.82$, $^{***}P = 3.0799E{-}22$, two-tailed paired Student's *t*-test. Source data are provided as a Source Data file.

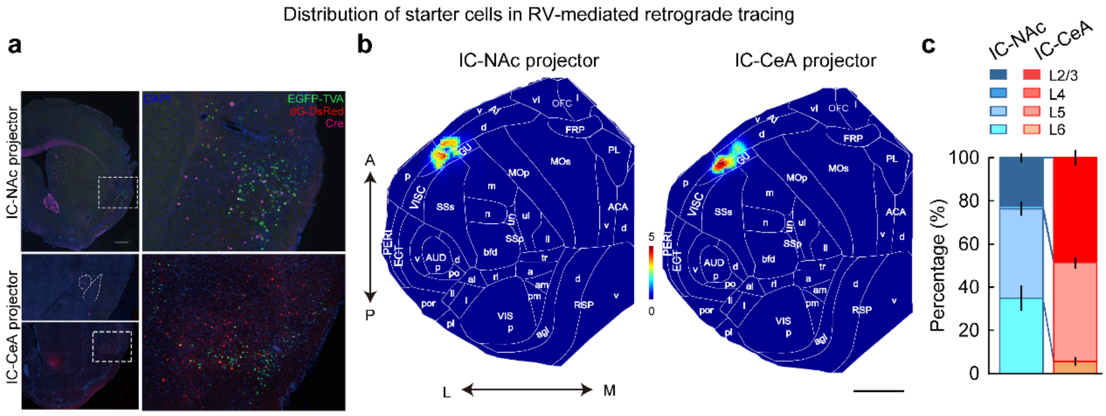

Supplementary Fig. 21. Characterization of starter cells for RV-mediated retrograde tracing of IC-CeA or IC-NAc projectors. a Representative images of virus expressed in the CeA and NAc, in addition to IC-CeA or IC-NAc projector starter cells. The purple immunofluorescent signal indicates the expression of Retro-AAV-Syn-Cre, the green signal shows the expression of EGFP-TVA, the red signal represents the expression of dG-DsRed, and the blue signal represents DAPI staining. Left: lower magnification images; right: higher magnification images showing the IC starter cells. Scale bar, 500 μm. **b** Distributions of starter cells, shown on flat two-dimensional maps, for RV-mediated retrograde tracing. Left: IC-NAc projectors; right: IC-CeA projectors. In both groups, the starter cells were mainly located in agranular insular area (AI) and gustatory area (GU). The colored scale is in reference to the normalized starter cell density. Scale bar, 1 mm. **c** Laminar distribution of starter cells for IC-CeA and IC-NAc projectors. IC-NAc projectors, n = 3 mice; IC-CeA projectors, n = 4 mice. Data are presented as mean values ± SEM and the error bar represents SEM. Source data are provided as a Source Data file.

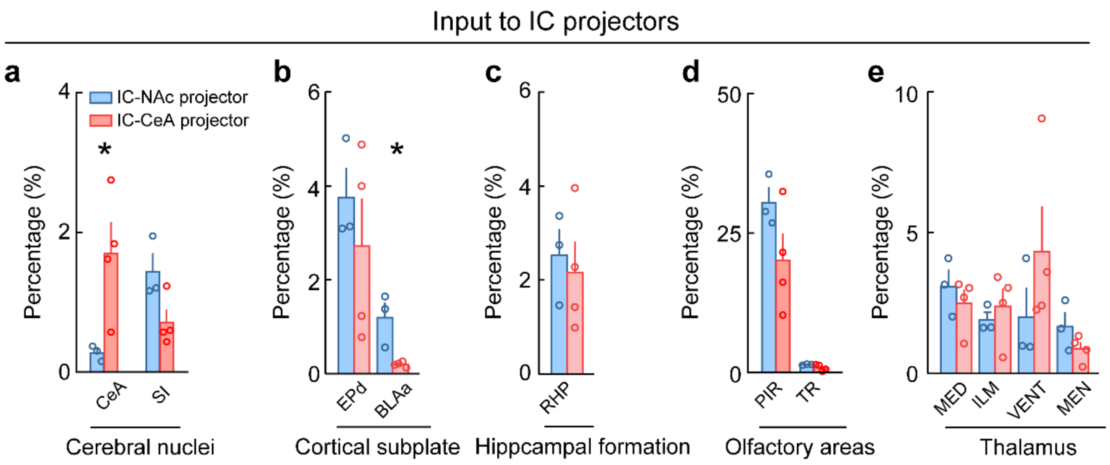

Supplementary Fig. 22. Quantification of labeled monosynaptic inputs to IC-CeA or IC-NAc projectors. The percentages of total inputs are shown. **a** SI, substantia innominata. **b** EPd, endopiriform nucleus, dorsal part; BLAa, basolateral amygdalar nucleus, anterior part. **c** RHP, retrohippocampal region. **d** PIR, piriform area; TR, postpiriform transition area. **e** MED, medial group of the dorsal thalamus; ILM, intralaminar nuclei of the dorsal thalamus; VENT, ventral group of the dorsal thalamus; MTN, midline group of the dorsal thalamus. IC-NAc projector, n = 3 mice; IC-CeA projectors, n = 4 mice. CeA: $t_{(5)} = 2.667$, $^{*}P = 0.0445$; SI: $t_{(5)} = 2.442$, $P = 0.0585$; EPd: $t_{(5)} = 0.7845$, $P = 0.4682$; BLAa: $t_{(5)} = 3.613$, $^{*}P = 0.0153$; RHP: $t_{(5)} = 0.4000$, $P = 0.7057$; PIR: $t_{(5)} = 1.718$, $P = 0.1465$; TR: $t_{(5)} = 1.583$, $P = 0.1743$; MED: $t_{(5)} = 0.7813$, $P = 0.47$; ILM: $t_{(5)} = 0.6186$, $P = 0.5633$; VENT: $t_{(5)} = 1.115$, $P = 0.3155$; MTN: $t_{(5)} = 1.546$, $P = 0.1829$; two-tailed unpaired Student's *t*-test. Data are presented as mean values ± SEM and the error bar represents SEM. Source data are provided as a Source Data file.

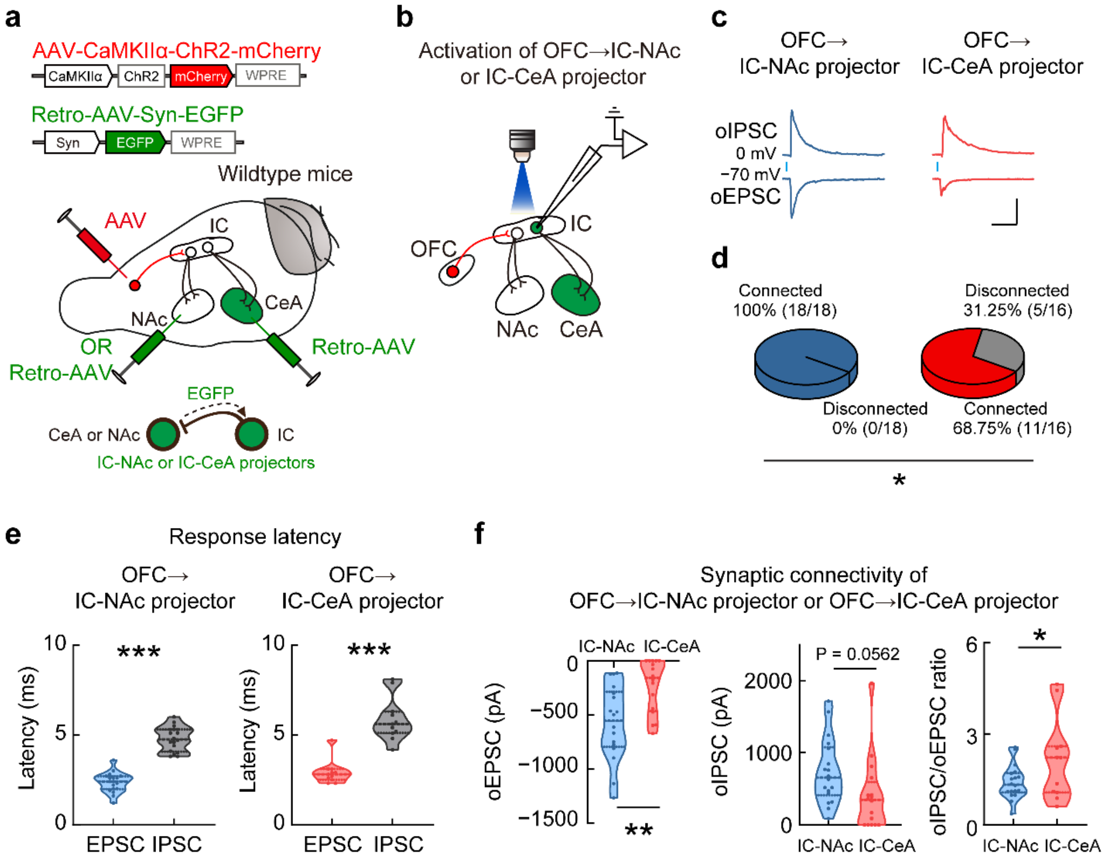

Supplementary Fig. 23. Electrophysiological verification of biased projections from the OFC to IC-NAc or IC-CeA projectors. a Experimental schematic for AAV injections. **b** Schematic for electrophysiological recordings from IC-NAc or IC-CeA projectors. **c** Representative traces of oEPSCs and oIPSCs, recorded from cells clamped at –70 and 0 mV, respectively, of the synapses from the OFC to IC-NAc or CeA projectors induced by photostimulation (5 ms, blue vertical bars). Scale bars, 100 ms, 100 pA. **d** Quantification of connectivity types (connected versus disconnected) in the projections of OFC → IC-NAc projectors and OFC → IC-CeA projectors. IC-NAc projectors, n = 18 neurons of 4 mice; IC-CeA projectors, n = 16 neurons of 4 mice. $^{*}P = 0.0157$; χ^2 Fisher's exact test. **e** Quantification of latency of synaptic connectivity on IC-CeA or IC-NAc projectors driven by activation of OFC inputs. IC-NAc projectors, n = 18 neurons of 4 mice; IC-CeA projectors, n = 11 neurons of 3 mice. Left: $t_{(17)} = 16.14$, $^{***}P = 9.6215\text{E}{-12}$; Right: $t_{(10)} = 10.64$, $^{***}P = 8.9477\text{E}{-07}$, two-tailed paired Student's *t*-test. **f** Quantification of current amplitudes of EPSC and IPSC and their ratios. IC-NAc projectors, n = 18 neurons of 4 mice; IC-CeA projectors, n = 16 neurons of 4 mice. Left: $t_{(32)} = 3.333$, $^{**}P = 0.0022$; Middle: $t_{(32)} = 1.982$, $P = 0.0562$; Right: $t_{(27)} = 2.068$, $^{*}P = 0.0483$, two-tailed unpaired Student's *t*-test. Source data are provided as a Source Data file.

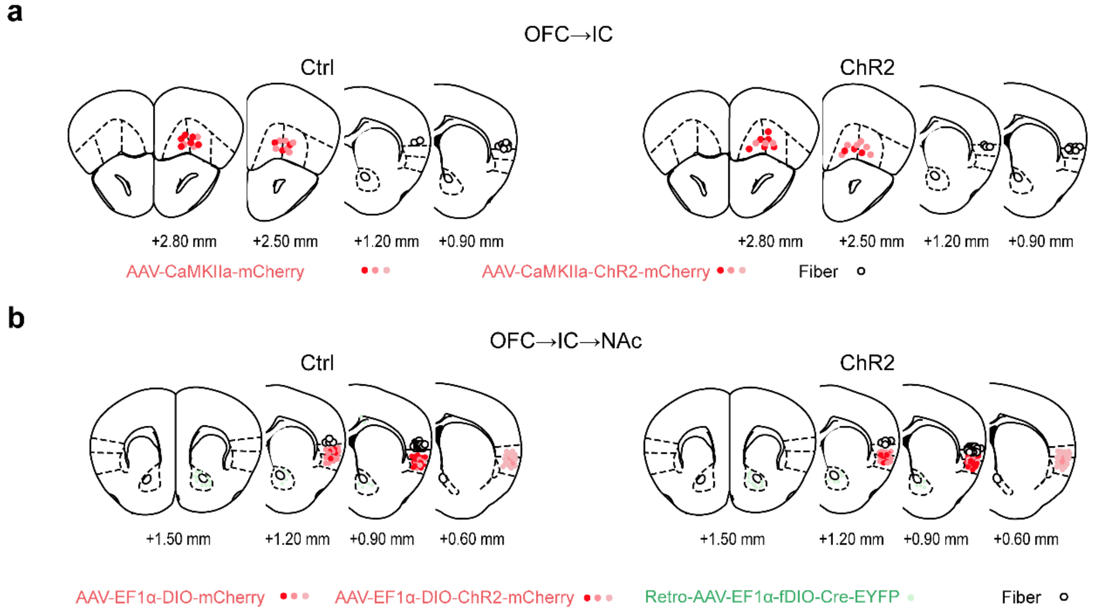

Supplementary Fig. 24. Histological confirmation of optical fiber placements for optogenetic activation of OFC→IC projection (a) or OFC→IC→NAc pathway (b), related to Fig. 7c–g. a Ctrl (mCherry), n = 9 mice; ChR2, n = 9 mice. **b** Ctrl (mCherry), n = 18 mice, ChR2, n = 20 mice.

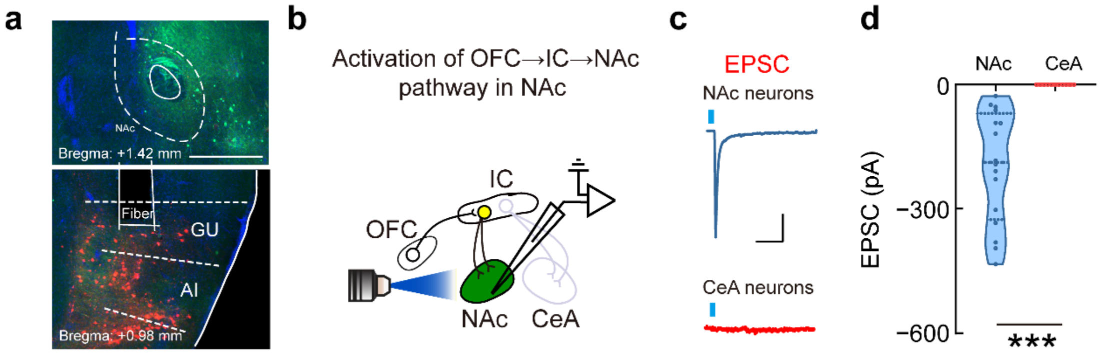

Supplementary Fig. 25. Histological and electrophysiological verifications of ChR2-mCherry expression in IC-NAc projectors dedicated to receiving long-range projections from the OFC. a Representative images of ChR2-mCherry expression (mCherry) in the IC from a mouse that received injections of the anterograde AAV1-EF1α-Flp into the OFC, that of the flippase-dependent retrograde AAV-EF1α-fDIO-Cre-EGFP into the NAc, and that of the Cre-dependent AAV-EF1α-DIO-ChR2-mCherry into the IC. Notably, sparse $EGFP^+$ cells were observed in the NAc, possibly reflecting a direct anterograde projection from the OFC to NAc. Scale bar, 500 µm. **b** Experimental schemes for electrophysiological verification of ChR2-mCherry expression in IC NAc projectors dedicated to receiving long-range projections from the OFC. **c** Representative traces of oEPSCs recorded from NAc (*upper*) or CeA (*lower*) neurons. Scale bars, 100 ms, 100 pA. **d** Statistical results are shown. IC to NAc: n = 16 neurons from 3 mice; IC to CeA: n = 11 neurons from 3 mice. $t_{(25)} = 4.777$, $^{***}P = 6.6436E{-}05$, two-tailed unpaired Student's *t*-test. Source data are provided as a Source Data file.

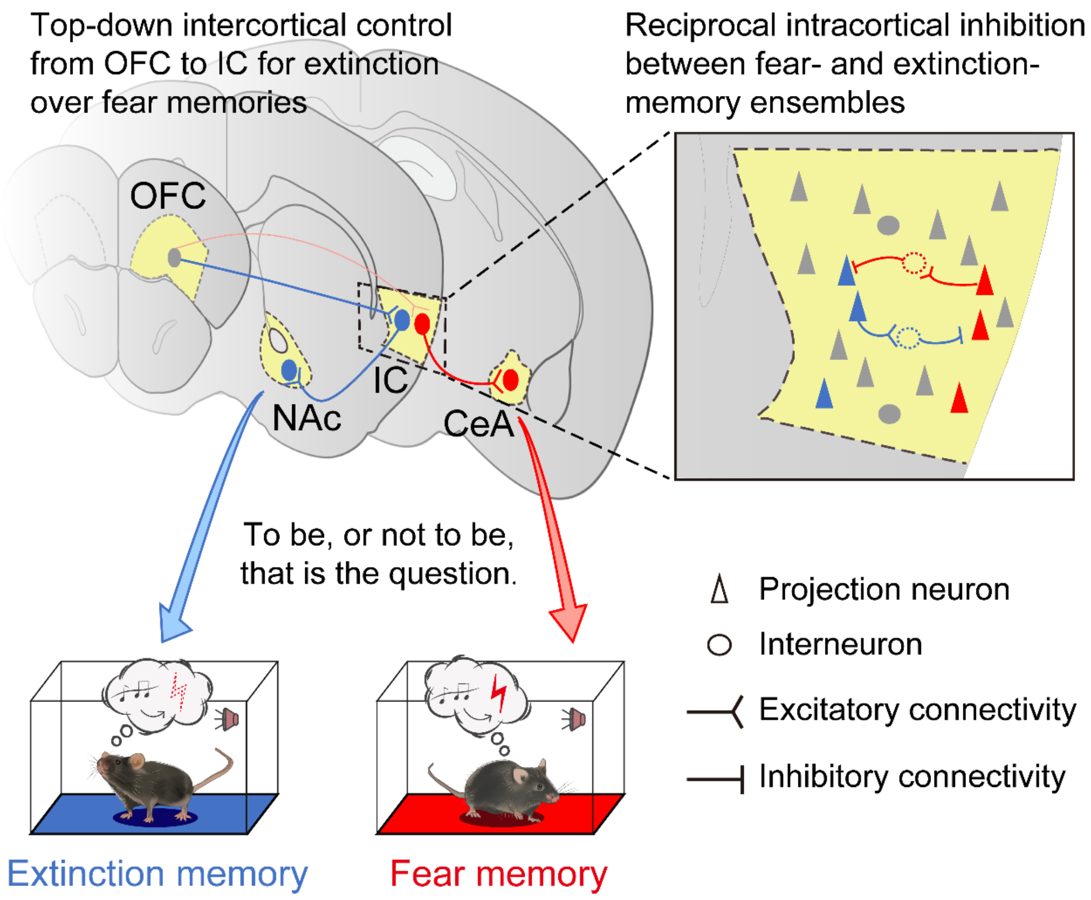

Supplementary Fig. 26. Scheme for cortical circuits as an executive gateway to decipher threat or extinction memory via distinct subcortical pathways. The well-adjusted activities of two distinct populations of IC neurons, defined by their differential long-range connectivities, coordinate respective fear and extinction memories for survival. IC-CeA and IC-NAc projectors encode fear and extinction memories, respectively. The reciprocal inhibitions of IC-CeA and IC-NAc projectors via local interneurons drive memory-guided behaviors in opposite directions, and their activities undertake distinct modifications during threat and extinction learning. Moreover, the OFC→IC→NAc circuit selectively engages extinction memory and thereby strengthens the specificity of distinct populations of IC neurons defined by their long-range connectivity. This figure was created from scratch with Adobe Illustrator (version 25.2.1, Adobe Inc., USA), Adobe Photoshop (version 22.3.1, Adobe Inc., USA), and Procreate (version 5.2, Savage Software Group Pty. Ltd., Australia).
