# Supplementary material for: Insular cortical circuits as an executive gateway to decipher threat or extinction memory via distinct subcortical pathways
Source: Nat Commun. 2022 Sep 21;13:5540. doi: 10.1038/s41467-022-33241-9 (PMC9492683; doi:10.1038/s41467-022-33241-9)
Supplement: Supplementary file 3 — Reporting Summary [file 41467_2022_33241_MOESM3_ESM.pdf]

Corresponding author(s): Tian-Le Xu, Wei-Guang Li, Siyu Zhang

Last updated by author(s): Jul 25, 2022

Reporting Summary

Nature Portfolio wishes to improve the reproducibility of the work that we publish. This form provides structure for consistency and transparency in reporting. For further information on Nature Portfolio policies, see our Editorial Policies and the Editorial Policy Checklist.

Statistics

For all statistical analyses, confirm that the following items are present in the figure legend, table legend, main text, or Methods section.

n/a	Confirmed	
☐	☒	The exact sample size (n) for each experimental group/condition, given as a discrete number and unit of measurement
☐	☒	A statement on whether measurements were taken from distinct samples or whether the same sample was measured repeatedly
☐	☒	The statistical test(s) used AND whether they are one- or two-sided<br>*Only common tests should be described solely by name; describe more complex techniques in the Methods section.*
☐	☒	A description of all covariates tested
☐	☒	A description of any assumptions or corrections, such as tests of normality and adjustment for multiple comparisons
☐	☒	A full description of the statistical parameters including central tendency (e.g. means) or other basic estimates (e.g. regression coefficient) AND variation (e.g. standard deviation) or associated estimates of uncertainty (e.g. confidence intervals)
☐	☒	For null hypothesis testing, the test statistic (e.g. F, t, r) with confidence intervals, effect sizes, degrees of freedom and P value noted<br>*Give P values as exact values whenever suitable.*
☒	☐	For Bayesian analysis, information on the choice of priors and Markov chain Monte Carlo settings
☒	☐	For hierarchical and complex designs, identification of the appropriate level for tests and full reporting of outcomes
☒	☐	Estimates of effect sizes (e.g. Cohen's d, Pearson's r), indicating how they were calculated

Our web collection on statistics for biologists contains articles on many of the points above.

Software and code

Policy information about availability of computer code

Data collection	ANY-maze 6.31 (Stoelting Co., USA)<br>Ethovision video tracking system (Noldus Information Technology, Netherlands)<br>Nikon A1R laser confocal microscope (Nikon, Japan)<br>Olympus VS120 (Olympus, Japan)<br>pCLAMP 10.5 (Molecular Devices, USA)<br>Zeiss Axio Scan. ZI (Zeiss, Germany)
Data analysis	ANY-maze (version 6.31, Stoelting Co., USA)<br>Noldus EthoVision XT (version 16.0, Noldus Information Technology, Netherlands)<br>GraphPad Prism (version 8.0.2, GraphPad Software, Inc., USA)<br>ImageJ 1.8.0 (NIH, USA)<br>MATLAB (R2014a, USA)<br>MiniAnalysis 6.0.1 program (Synaptosoft Inc., USA)<br>Office 2019 (Microsoft, USA)<br>Origin 9.5 Software (OriginLab Corporation, Northampton, MA)<br>pCLAMP 10.5 (Molecular Devices, Sunnyvale, California, USA)

For manuscripts utilizing custom algorithms or software that are central to the research but not yet described in published literature, software must be made available to editors and reviewers. We strongly encourage code deposition in a community repository (e.g. GitHub). See the Nature Portfolio guidelines for submitting code & software for further information.

Data

Policy information about availability of data

All manuscripts must include a data availability statement. This statement should provide the following information, where applicable:

- Accession codes, unique identifiers, or web links for publicly available datasets
- A description of any restrictions on data availability
- For clinical datasets or third party data, please ensure that the statement adheres to our policy

All the source data are provided with this paper and can be accessed without restrictions. No new code was developed in the study.

Field-specific reporting

Please select the one below that is the best fit for your research. If you are not sure, read the appropriate sections before making your selection.

☒ Life sciences ☐ Behavioural & social sciences ☐ Ecological, evolutionary & environmental sciences

For a reference copy of the document with all sections, see nature.com/documents/nr-reporting-summary-flat.pdf

Life sciences study design

All studies must disclose on these points even when the disclosure is negative.

Sample size	No statistical methods were used to pre-determine sample sizes but our sample sizes are similar to those reported in previous publications (e.g., Nat Commun 7: 13770, 2016; Sci Adv 4: eaau3075, 2018)
Data exclusions	No data points were excluded.
Replication	Yes; all attempts at replication were successful.
Randomization	Randomization was used to assign all experimental groups without bias based on any covariates.
Blinding	All experiments except eletrophysiological experiment were performed by raters blinded to the experimental groups. For electrophysiological experiment, preparation of the brain slices used for electrophysiological recordings was performed by an investigator with knowledge of the identity of the experimental group, while the collection and analysis of electrophysiological data were done independently by an experienced experimenter in a blind manner. All behavior experiments were controlled by computer systems, and data were collected and analyzed in an automated and unbiased way.

Reporting for specific materials, systems and methods

We require information from authors about some types of materials, experimental systems and methods used in many studies. Here, indicate whether each material, system or method listed is relevant to your study. If you are not sure if a list item applies to your research, read the appropriate section before selecting a response.

Materials & experimental systems

n/a	Involved in the study	
☐	☒	Antibodies
☒	☐	Eukaryotic cell lines
☒	☐	Palaeontology and archaeology
☐	☒	Animals and other organisms
☒	☐	Human research participants
☒	☐	Clinical data
☒	☐	Dual use research of concern

Methods

n/a	Involved in the study	
☒	☐	ChIP-seq
☒	☐	Flow cytometry
☒	☐	MRI-based neuroimaging

Antibodies

Antibodies used	The primary antibodies used were: rabbit anti-c-fos (Cell Signaling Technology, USA, cat. # 2250) and rabbit anti-Cre (Cell Signaling Technology, USA, cat. # 15036).<br>The fluorophore-conjugated secondary antibodies used were: Donkey anti-Rabbit IgG (H+L) Highly Cross-Adsorbed Secondary Antibody, Alexa Fluor™ 488 (ThermoFisher Scientific, USA; cat. # A21206) and Donkey anti-Rabbit IgG (H+L) Highly Cross-Adsorbed Secondary Antibody, Alexa Fluor™ 647 (ThermoFisher Scientific, USA; cat. # A31573).
Validation	The primary antibody of rabbit anti-c-fos has been validated by many publications with immunohistochemistry techniques in mice such as Nat Commun 13: 998, 2022; Mol Psychiatry, doi: 10.1038/s41380-022-01684-7, 2022.

The primary antibody of rabbit anti-Cre has been validated by many publications with immunohistochemistry techniques in mice such as Nat Commun 10: 2906, 2019; Nat Commun 9: 1420, 2018.

Animals and other organisms

Policy information about studies involving animals; ARRIVE guidelines recommended for reporting animal research

Laboratory animals	Mice:<br>Most experiments were done on the C57BL/6J mice, purchased from Shanghai Laboratory Animal Center (SLAC) at the Chinese Academy of Sciences (Shanghai, China).<br>The following mice are available at the Jackson Laboratory, Bar Harbor, Maine, USA: Fos2A-iCreER (TRAP2) (stock #030323).<br>Mice of 8–12 weeks old of age were used for all the experiments. Only male mice were used. All mice were bred in specific pathogen-free laboratory animal facilities under standard conditions with temperatures of 21–23°C, 40–60% humidity, and a 12-h light/dark cycle with rodent chow and water provided ad libitum. The information is provided in the Methods section.
Wild animals	The study did not involve wild animals.
Field-collected samples	The study did not involve samples collected from the field.
Ethics oversight	All of the animal procedures were approved by the Animal Ethics Committee of Shanghai Jiao Tong University School of Medicine and by the Institutional Animal Care and Use Committee (Department of Laboratory Animal Science, Shanghai Jiao Tong University School of Medicine; Policy Number DLAS-MP-ANIM. 01–05).

Note that full information on the approval of the study protocol must also be provided in the manuscript.
